# The plant early recombinosome: a high security complex to break DNA during meiosis

**DOI:** 10.1007/s00497-024-00509-7

**Published:** 2024-09-27

**Authors:** Nahid Rafiei, Arnaud Ronceret

**Affiliations:** Department of Plant Molecular Biology, Instituto de Biotecnología (IBT), Universidad Nacional Autónoma de México (UNAM), Cuernavaca, Morelos México

**Keywords:** Meiosis, Early recombinosome, Double strand break formation, SPO11 complex, Axial elements

## Abstract

**Key message:**

The formacion of numerous unpredictable DNA Double Strand Breaks (DSBs) on chromosomes iniciates meiotic recombination. In this perspective, we propose a ‘multi-key lock’ model to secure the risky but necesary breaks as well as a ‘one per pair of cromatids’ model for the topoisomerase-like early recombinosome.

**Abstract:**

During meiosis, homologous chromosomes recombine at few sites of crossing-overs (COs) to ensure correct segregation. The initiation of meiotic recombination involves the formation of DNA double strand breaks (DSBs) during prophase I. Too many DSBs are dangerous for genome integrity: if these DSBs are not properly repaired, it could potentially lead to chromosomal fragmentation. Too few DSBs are also problematic: if the obligate CO cannot form between bivalents, catastrophic unequal segregation of univalents lead to the formation of sterile aneuploid spores. Research on the regulation of the formation of these necessary but risky DSBs has recently advanced in yeast, mammals and plants. DNA DSBs are created by the enzymatic activity of the early recombinosome, a topoisomerase-like complex containing SPO11. This opinion paper reviews recent insights on the regulation of the SPO11 cofactors necessary for the introduction of temporally and spatially controlled DSBs. We propose that a ‘multi-key-lock’ model for each subunit of the early recombinosome complex is required to secure the formation of DSBs. We also discuss the hypothetical implications that the established topoisomerase-like nature of the SPO11 core-complex can have in creating DSB in only one of the two replicated chromatids of early prophase I meiotic chromosomes. This hypothetical ‘one per pair of chromatids’ DSB formation model could optimize the faithful repair of the self-inflicted DSBs. Each DSB could use three potential intact homologous DNA sequences as repair template: one from the sister chromatid and the two others from the homologous chromosomes.

**Supplementary Information:**

The online version contains supplementary material available at 10.1007/s00497-024-00509-7.

## Introduction

Meiotic crossing-overs (CO) are genomic sites where DNA molecules reshuffle to allow the formation of new recombined chromosomes (Zickler and Kleckner 2023). The molecular model for meiotic recombination involves the initial formation of DNA double strand breaks (DSBs) (Szostak et al. 1983). Due to the complexity of chromosomal programs during meiosis, the mechanisms of DSB and CO formation are still not completely understood (Jones et al. 2024). DSBs are formed on unsynapsed meiotic chromosomes after replication. In addition to meiotic cohesin complexes that maintain sister chromatid cohesion, the meiotic specific axial element (AE) of the synaptonemal complex also organizes the replicated chromosomes along linear structures from telomere to telomere (Mercier et al. 2015; Zickler and Kleckner 2023). AEs are later transformed into Lateral Elements (LEs) of the Synaptonemal Complex (SC) that connect and bring together both synapsed homologous chromosomes to a proximity of about 200 nm (Mercier et al. 2015; Zickler and Kleckner 2023). Various components of the SC have effects on the regulation of formation and positioning of DSBs and COs (Zickler and Kleckner 2023). Compared to COs, DSBs are usually produced in excess. The designation of the DSBs that will form the few COs involves layers of control at several intermediate stages (Mercier et al. 2015; Zickler and Kleckner 2023). Therefore, there is an incomplete correlation between DSB and CO patterns (Serrentino and Borde 2012; Mézard et al. 2015; Tock and Henderson 2018). It seems that the determinants of recombination landscape are progressively imposed during early (leptotene), middle (zygotene) and late (pachytene) stages, involving both stabilizing (pro-CO) and dissolving (anti-CO) pathways (Mercier et al. 2015). These several layers of temporal control during the recombination process are also combined with control at the spatial level (reviewed in Mézard et al. 2015; Tock and Henderson 2018 and Ziolkowski et al. 2019).

In plants, recombination is not spatially uniform along the chromosome and some highly recombinogenic (hotspot) and low recombinogenic (coldspot) regions have been identified (reviewed in Tock and Henderson 2018; Okagaki et al. 2018; Fernandes et al. 2019). Most Angiosperms show a U shape distribution of DSBs and COs along chromosomes, with higher level near the peri-telomeric regions, where gene density is also the highest (Li et al. 2015; He et al. 2017; Choi et al. 2018). It is still unclear how the uneven distribution of genes and repeats shape the recombination profile in Angiosperms, since these features also covary with various epigenetic marks (Yelina et al. 2015; Choi et al. 2018; Zhao et al. 2021). However, this U shape recombinogenic pattern is also present in holocentric plants with centromeres all along the length of the chromosome, suggesting that this pattern is not due to centromere composition but could rather reflect an asymmetrical recombinogenic process starting at the telomeres (Castellani et al. 2024). Whether or not this U shape recombinogenic profile is also observed in the chromosomes of basal plants with evenly distributed genes and repeats such as some bryophytes (Lang et al. 2018; Li et al. 2020)*,* ferns (Huang et al. 2022) and lycophytes (Li et al. 2024), has, to our knowledge, not yet been analyzed. In Arabidopsis, hotspots usually occur outside genic regions, in low nucleosome occupancy regions of promoters and terminators, which have high chromatin accessibility and low DNA methylation (Choi et al. 2013; Choi and Henderson 2015; Choi et al. 2018; Tock and Henderson 2018). In hexaploid wheat, though most recombination hotspots are also found in promoters (Darrier et al. 2017), homeologous exchanges usually occur inside genic regions (Zhang et al. 2020), suggesting that several levels of control could dictate CO positioning in polyploid species. Though, some specificities are observed, common chromosomal and DNA characteristics determine the positioning of meiotic recombination sites in several plant species (Wang et al. 2022a). In Arabidopsis and maize, the limitation of CO in centromeres and pericentromeric regions containing repetitive DNA does not seem to be due to a complete absence of DSBs (He et al. 2017). The repression of CO in centromeres also occurs after DSB formation for the resolution of these breaks into NCO instead of CO (Fernandes et al. 2019; Naish et al. 2021; Wang et al. 2022a, Fernandes et al. 2024). In addition to the progressive determination of DSB into CO (reviewed in Mézard et al. 2015; Zickler and Kleckner 2023), there is special control of the positioning and interspacing of most COs based on CO interference. For reviews of the general aspect of plant recombination models and CO patterning, please see Mercier et al. 2015; Wang and Copenhaver 2018; Gutiérrez-Pinzón et al. 2021; Rafiei and Ronceret 2023; Morgan et al. 2024).

The analysis of the initial step of meiotic recombination presents several challenging aspects that we would like to review and emphasize here:

*First*, the progression of meiosis is very dynamic (Ronceret et al. 2007; Ronceret and Pawlowski 2010; Prusicki et al. 2019). Few model organisms offer synchronized meiotic stages where only one step of recombination occurs, as it does in maize meiotic anthers (Nan et al. 2011; Nelms and Walbot 2019).

*Second,* the meiotic process in male and female meiosis is usually separated by surrounding somatic tissues. Male and female meiosis typically have different rates of recombination: a phenomenon known as heterochiasmy (Sardell and Kirkpatrick 2020). In plants, meiotic recombination has mainly been studied in anthers, but new techniques also allow analysis of female meiosis and heterochiasmy (Escobar-Guzmán et al. 2015, Galvan-Gordillo et al. 2020, Capilla-Pérez et al. 2021). In mammals, female meiotic prophase occurs during the fetal stage, making its analysis particularly difficult in human (Gray and Cohen 2016).

*Third,* the molecular process of homologous recombination occurs in a context of replicated chromosomes. Four homologous DNA molecules are present during meiotic prophase (Zickler and Kleckner 1999; Mercier et al. 2015). This mix of similar molecules is difficult to study using traditional biochemical, cytogenetic and, in the case of sister chromatids, even by genomic techniques.

*Fourth,* the SPO11 complex acts as an enzyme making only one endonuclease catalytic reaction (Keeney and Kleckner 1995; Hartung et al. 2007; Brinkmeier et al. 2022). The catalytic tyrosines of a SPO11 dimer initiate nucleophilic attacks of the phosphodiester nucleotide bound and create a DSB with two-nucleotides 5′ overhangs (Liu et al. 1995). SPO11 stays covalently linked to the 5′ overhang of the DNA molecule it has cut (Keeney and Kleckner 1995). So, the number of DSBs formed on each meiocyte depends on the number of the SPO11 protein complexes, also known as the early recombinosomes (Blat et al. 2002). The broken ends are precisely repaired by the process of homologous recombination using an intact homologous DNA molecule (Zickler and Kleckner 2023). DSBs create DNA ends that are degraded from 5′ to 3′ with a combination of endonucleases and exonucleases, a process called DNA 5′ end resection, to produce single strand DNA 3′ overhangs (Garcia et al. 2011). The nucleolytic degradation of the DSB DNA ends release the SPO11 proteins in the form of SPO11-oligonucleotides, that can be used to sequence and identify genome-wide DSB locations in yeast, mammals and plants (reviewed in Hwang and Hunter 2011; Tock and Henderson 2018; Jing et al. 2019).

*Fifth,* between each nuclear meiocyte, the position of DSBs is variable, random and unpredicSupplementary Table. Characterization of the concomitant occurrence of hundreds of stochastic DSBs in the genome affecting all chromosomes during prophase I requires whole genome approaches (Cooper et al. 2016; Tock and Henderson 2018; Jing et al. 2019; Sun et al. 2019). The mechanism of concerted close ‘double cuts’, involving about 20% of the DSBs described in *S. cerevisiae,* and its implications for Spo11 preference for DNA bending motif (Johnson et al. 2021; Prieler et al. 2021) has not yet been investigated in plants.

*Sixth,* the development of a DSB into a CO is difficult to study because not all DSBs will form a CO, though all COs derive from a DSB. Only a small fraction of DSBs (less than 10% in Arabidopsis) will generate a CO. Most of the DSBs that do not form a CO are repaired using either the sister chromatid as an intact template or forming a small patch of recombined region known as Non-CrossOver (NCO) between homologous chromosomes (Lao and Hunter 2010; Mercier et al. 2015).

*Seventh,* the random nature of the DNA breaks along large chromosomes makes it difficult to analyze using regular molecular biology and biochemical methods, since the individual broken DNA substrate sites are difficult to define. For many years, it was difficult to analyze DSBs and COs, except in a few hotspot genomic sites more prone than other genomic regions to form DSBs and ultimately COs (Fu et al. 2001; Yandeau-Nelson et al. 2005, 2006; Drouaud et al. 2013). New techniques of whole genome sequencing and chromatin immunoprecipitation now allow whole genome mapping of DSBs and COs with high resolution (review in Tock and Henderson 2018). The analysis of meiotic recombination at the whole genome level, first realized in budding yeast (Mancera et al. 2008), allows a more global understanding of meiotic recombination, not only in Arabidopsis (Choi et al. 2018; Lian et al. 2022) but also in several flowering plants (Brazier and Glémin 2022) with big genomes such as maize (Li et al. 2015; He et al. 2017, Kanian et al. 2018) and wheat (Darrier et al. 2017; Zhang et al. 2020).

In this opinion paper, we want to discuss possible implications of the latest understanding of the machinery known as the early recombinosome, involved in generating DSBs during plant early meiotic prophase.

## The plant SPO11-independent pathways for DSB formation

During meiosis, the vast majority of DSBs is dependent on the SPO11 complex. However, few SPO11-independent pathways have been described. This SPO11-independent residual DSB formation is difficult to observe in wild type situations since it is masked by numerous SPO11-dependent DSB sites. Therefore, these minor DSB formation pathways are difficult to observe except in *spo11-like* mutants. In mouse, a substantial percentage of meiotic recombination initiation is due to MULE-MuDR DNA transposons excisions (Yamada et al. 2017). The DNA transposons of the Mutator (Mu) and Mutator-like elements (Mule) family can create DSBs when excising (Lisch et al. 2013; Liu and Wessler 2017). In *C. elegans,* the Mos1 transposon excision can be used to form unique CO sites in *spo-11* mutant background (Altendorfer et al. 2020). In maize *spo11-1* and *phs1* mutants, most meiocytes behave as do DSB mutants of other species, though 10% of meiocytes have atypical phenotypes such as diffuse TUNEL labelling (detecting DSB ends), non-homologous synapsis and the presence of one to two residual bivalents (Pawlowski et al. 2004; Ronceret et al. 2009; Ku et al. 2020). As these maize DSB mutants come from Mutator active populations, we speculate that infrequent DSBs induced by germinal Mu excisions might explain these unexpected phenotypes. In maize, the presence of Mutator is associated with higher meiotic recombination rates (Yandeau-Nelson et al. 2005; Liu et al. 2009). In other plant species such as Arabidopsis, Helitron transposons are also found around the hottest DSB hotspots (Choi et al. 2018; Underwood and Choi 2019). However, in plants the suspected role of transposon excisions in the creation of some meiotic DSBs has not yet been directly analyzed.

SPO11-independent fragmentation of chromosomes during meiosis has also been observed in the Arabidopsis *mei1* (Grelon et al. 2003) and *xri1* mutants (Dean et al. 2009). These pathways were usually explained as involved in premeiotic replication errors leading to SPO11-independent DSB formation (Grelon et al. 2003; Dean et al. 2009). Arabidopsis *mei1* (Grelon et al. 2003) has now been identified as a topoisomerase II (TOP2) Binding Protein1 (TopBP1) mutant (Parra-Nunez et al. 2021). While the budding yeast Top2 protein is important in meiosis and mediates CO interference (Zhang et al. 2014), the Arabidopsis TOP2 ortholog is involved in the resolution of entangled chromosomes and interlocks, for meiotic DSB repair progression but not for CO formation (Martinez-Garcia et al. 2018, 2021). The role of these minor SPO11-independent DSB formation pathways remains poorly explored in plants.

## The SPO11 core complex is related to topoisomerase VI and requires several cofactors to form the vast majority of DSB

**Fig. 1:**
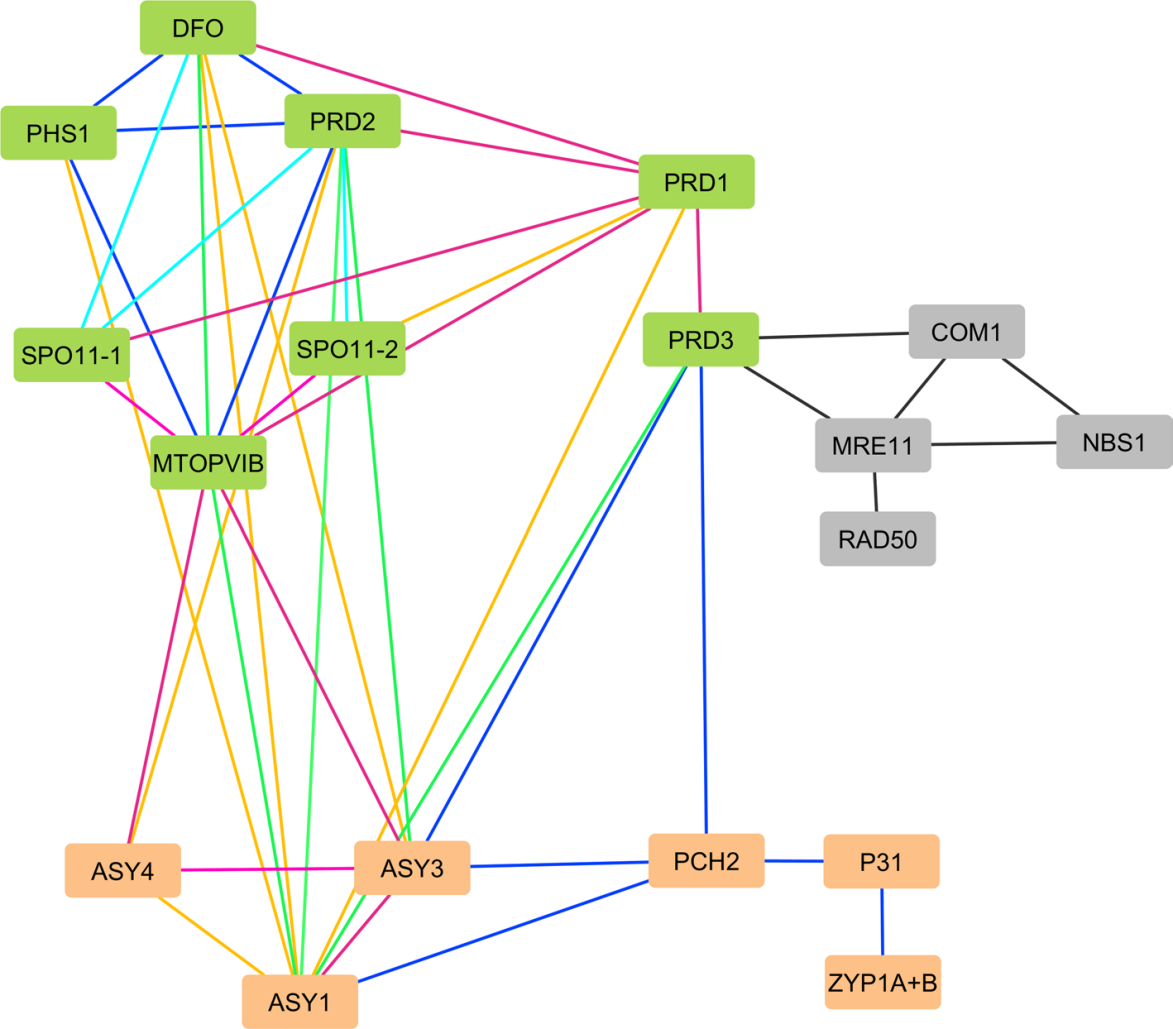
The Arabidopsis early recombinosome protein-protein interaction network observed using various assays. Protein-protein interactions identified through the Yeast Two-Hybrid (Y2H) assay; a high-throughput molecular biology technique aimed at discerning such protein interactions within their native environment. Interactions detected in selective media lacking leucine, tryptophan, and histidine (LWH), as well as adenine (LWHA), are represented by light blue and dark blue lines, respectively. In addition, the assessment of independent protein-protein interactions in plant systems using the Bimolecular Fluorescence Complementation (BiFC) method is represented by orange lines. Instances where interactions are confirmed by both LWH and BiFC are shown by a green line, whereas those corroborated by LWHA and BiFC are depicted with purple lines. In the diagram, the green boxes denote involvement in the formation of double-strand breaks (DSBs), while the orange boxes signify components of axial elements. Furthermore, the grey boxes represent the MRN complex, crucial for both mitotic and meiotic DSB resection, and its interaction with COM1, illustrated by black lines. These interactions were described in Arabidopsis in Vrielynck et al. (2021)

Most understanding of molecular mechanisms for meiotic recombination initiation comes from the yeast models *Saccharomyces cerevisiae* (Claeys Bouuaert et al. 2021a and 2021b) and *Schizosaccharomyces pombe* (Hyppa et al. 2022). In the budding yeast *S. cerevisiae,* in addition to the major catalytic Spo11 subunit (Keeney et al. 1997; Bergerat et al. 1997), it is known that nine other proteins are required for the formation of DSBs (reviewed in Yadav and Claeys Bouuaert 2021). The budding yeast early recombinosome is composed of the core complex (Spo11, Rec102, Rec103 and Ski8), associated to the RMM complex (Rec114, Mei4, Mer2) and the MRX complex (Mre11, Rad50 and Xrs2) (Claeys Bouuaert et al. 2021a, b). Names of orthologous proteins, that can change from species to species, are given in Supplementary Table 1. Various insights have also shown that the overall structure of the early recombinosome machinery is conserved, but that some variation exists between species (de Massy 2013; Arter and Keeney 2024). The Spo11 protein is the most conserved protein of the early recombinosome complex while the additional subunits evolve more rapidly (De Massy 2013; Brinkmeier et al. 2022; Thangavel et al. 2023). Recently, a function for these additional proteins has been shown in DNA-protein condensate formation due to phase transition of these unstructured domain-containing cofactor proteins (Claeys Bouuaert et al. 2021b). Most early recombinosome proteins interact with each other and a subgroup can link with some axial elements as observed in Arabidopsis and maize (Vrielynck et al. 2021; Wang et al. 2023b) (Fig. 1).

### In plants, SPO11-1 and SPO11-2 are both indispensable for DSB formation while SPO11-3 has a divergent function in somatic endoreduplication

In plants, SPO11 is part of a three member gene family with A-subunit topoisomerase VI homology (Grelon et al. 2001). SPO11-1 and SPO11-2 are both required for DSB formation in Arabidopsis (Grelon et al. 2001; Stacey et al. 2006; Hartung et al. 2007), rice (Fayos et al. 2020), maize (Ku et al. 2020; Li et al. 2022), and wheat (Da Ines et al. 2020; Benyahya et al. 2020; Hyde et al. 2023) (Supplementary Table 1). SPO11-2 is present in all plant lineages while SPO11-1 is not present in algae or the bryophyte *Marchantia polymorpha* (Thangavel et al. 2023). The other plant somatic type VI topoisomerases of the SPO11 family such as SPO11-3/BIN5, that also form a complex with a somatic counterpart TOPVI/BIN3, are essential for sporophytic plant development but dispensable for meiosis (Hartung et al. 2002; Yin et al. 2002). In Arabidopsis and upland cotton, SPO11-3 is involved in endoreduplication and is supposed to be essential for the decatenation of replicating chromosomes during the cell cycle (Sugimoto-Shirasu et al. 2005; Kirik et al. 2007; Wei et al. 2019). In rice, two additional SPO11 genes named SPO11-4 and SPO11-5 are present, with SPO11-4 having DSB forming capacities (An et al. 2011). While analysis of RNAi *Os-spo11-4* knock-down plants seemed to indicate that OsSPO11-4 could produce male meiotic defects (An et al. 2011), these effects were probably unspecific, as analysis of CRISPR null *Os-spo11-4* alleles showed that OsSPO11-4 is not involved in meiotic recombination (Fayos et al. 2020) (Supplementary Table 1). The comparison of the specific domains of these other topoisomerase VI complex components and whether they can interact with the other members of the early meiotic recombinosome has not been tested. However, this analysis could bring valuable comparative biochemical and mechanistic information about the specificity of the early recombinosome in relation to other somatic topoisomerase VI complexes.

Interestingly, the functional conservation of the SPO11-1 gene from various Angiosperm species was tested by interspecific complementation experiments (Sprink and Hartung 2021). It was originally found that divergent SPO11-1 genes were only able to complement the Arabidopsis *spo11-1* mutant using the SPO11-1 coding sequence from close relatives such as Brassica or papaya, but not rice (Sprink and Hartung 2021). It is now known that this complementation can also work between more distant relatives, since wheat TaSPO11-1-5D can complement the rice *spo11-1* mutant, as well as the Arabidopsis *spo11-1* mutant (Da Ines et al. 2020). Whether or not such complementation strategies will also work for more divergent components of the early recombinosome such as PRD3, PHS1 and DFO will be interesting to test in the future.

In mammals, the unique SPO11 gene (Romanienko and Camerini-Otero 2000) has α and β isoforms (Bellani et al. 2010; Cesari et al. 2020), resulting in a dramatic influence on the localization of the SPO11 complex since these isoforms are respectively found on male sexual chromosomes (α) and autosomes (β) (Kauppi et al. 2011). In several plants, SPO11-1 and SPO11-2 express different isoforms (Sprink and Hartung 2014). In maize, SPO11-1 has two isoforms as observed in mammals, called by analogy α and β isoforms (Ku et al. 2020) (Supplementary Table 1). The role of these two SPO11-1 isoforms is still not clear in maize; maize does not have sexual chromosomes but can sometimes transmit additional small B chromosomes (Blavet et al. 2021). These isoforms show peak transcriptional expression timing differences but whether the two isoforms can bind with different genomic regions is still unknown (Ku et al. 2020). Localization of maize SPO11-1 shows that a subset of foci are associated with the chromosomal axis and that the number of these axis-associated foci correspond to the number of DSBs formed in maize male meiocytes (Ku et al. 2020).

### MTOPVIB, the B subunit of the topoisomerase core complex, is necessary for SPO11-1 and SPO11-2 heterodimer formation

The function of the B subunit of the SPO11 core complex, named MTOPVIB in plants, is necessary for DSB formation in Arabidopsis (Vrielynk et al. 2016), rice (Xue et al. 2019), maize (Jing et al. 2020) and barley (Steckenborn et al. 2023). In Arabidopsis MTOPVIB is associated with the SPO11-1/SPO11-2 heterodimer that forms during the early recombinosome core-complex (Vrielynk et al. 2016; Tang et al. 2017; Chen et al 2024). In mammals, the B subunit is known as TOPVIBL and is also necessary for DSB formation (Robert et al. 2016a, 2016b). Mice TOPVIBL can interact only with SPO11β (and not SPO11α) (Robert et al. 2016a, b) also directly interacts with REC114 to regulate the timing of DSB formation (Nore et al. 2022). In fission yeast, this subunit is known as Rec6 (Lin and Smith 1994; Robert et al. 2016b) (Supplementary Table 1). In budding yeast, the B subunit corresponds to two DSB factors known as Rec102 and Rec104 (Kee et al. 2004; Claeys Bouuaert et al. 2021a). Structural modeling of the budding yeast SPO11 core complex shows that it maintains the global organization of type VI topoisomerase (Claeys Bouuaert et al. 2021a, b). The Arabidopsis mTOPVIB, that contains a degenerate GHKL domain usually important for ATP interaction (Vrielynk et al. 2016), has lost its ability to bind and hydrolyze ATP (Chen et al 2024). Manipulation of meiotic recombination has tremendous potential for agriculture (Taagen et al. 2020; Epstein et al. 2023). Interestingly, the fusion of MTOPVIB with Cas9, associated with the expression of a guide RNA, is not able to increase the frequency of CO in the targeted regions. This indicates that this is not an appropriate strategy to direct recombination in plants (Yelina et al. 2022), in contrast to results in budding yeast with SPO11 fusions proteins (Peciña et al. 2002; Sarno et al. 2017).

### In plants, the SKI8 protein and the MRN complex are not required for DSB formation

*S.cerevisiae* Ski8/Rec103 has a role in DSB formation in addition to its role in the SKI complex involved in the degradation of cleaved mRNAs without a stop codon (Gardiner et al. 1997; Arora et al. 2004). In Arabidopsis, the SKI8/VIP3 homolog is not required for DSB formation (Jolivet et al. 2006), even if its role as an SKI complex subunit is conserved, as demonstrated by the functional complementation of the *Atvip3* mutant by the ScSki8 gene (Dorcey et al. 2012).

The yeast Mre11-Rad50-Xrs2 (MRX) complex or its homolog called MRE11-RAD50-NBS1 (MRN) in plants and animals is one of the essential factors for the rapid identification of all kinds of DSBs formed in the genome during mitosis and meiosis (reviewed in Kieffer and Lowndes 2022). In budding yeast, the MRX complex is part of the early recombinosome and required for DSB formation (reviewed in Yadav and Claeys Bouuaert 2021). In plants, the MRN complex, formed by MRE11 (Puizina et al. 2004; Samanić et al. 2013; Ji et al. 2013; Nair et al. 2021), RAD50 (Bleuyard et al. 2004, Perez et al. 2011) and NBS1 (Waterworth et al. 2007) associated with COM1/CtIP (Uanschou et al. 2007; Ji et al. 2012) is dispensable for meiotic DSB formation but is required for mitotic and meiotic DSB resection, the first step in the DSB repair by homologous recombination. The dissociation of function between the early recombinosome and the MRN complex is well established in other plant and animal models, making the budding yeast (and *C.elegans*) MRX requirement for DSB formation an exception rather than the rule (Yadav and Claeys Bouuaert 2021). In Arabidopsis, the early recombinosome is still able to interact via PRD3 with the MRN complex, probably to facilitate the coordinated processing of DSBs once they are formed (Vrielynck et al. 2021). SPO11-1 and PRD3 show dynamic foci during Arabidopsis meiotic prophase I (Lambing et al. 2023) and it is possible that this reflects a dissociation between their role in DSB formation and association with members of the MRN complex. It will be interesting to determine the high resolution localization of the plant MRN on chromatids to test if it is also associated with axial elements during meiotic prophase I.

### PRD1 and PRD3 coordinate the association of various DSB factors

PUTATIVE RECOMBINATION DEFECT 1 (PRD1) was initially identified as an essential DSB factor in Arabidopsis (de Muyt et al. 2007). PRD1 shows limited conservation with the mammalian Mei1 protein, which is also essential for meiotic DSB formation (Libby et al. 2003) (Supplementary Table 1). PRD1 orthologs are essential for meiotic DSB formation in rice (Shi et al. 2021) and maize (Wang et al. 2022a, b), underlying the conserved role of this essential DSB factor. AtPRD1 can directly interact with AtSPO11-1 (de Muyt et al. 2007; Shingu et al. 2010; Tang et al. 2017). Yeast Two-Hybrid assays do not show a direct interaction between PRD1 with SPO11-2 (de Muyt et al. 2007; Vrielynck et al. 2021) but Biomolecular Fluorescence Complementation (BiFC) assay does show this interaction (Vrielynck et al. 2021) (Fig. 1). AtPRD1 also directly interacts with MTOPVIB, PRD2, PRD3 and DFO (Tang et al. 2017; Vrielynck et al. 2021) (Fig. 1) making it a central hub for connecting the different DSB forming factors. In rice, PRD1 is involved in spindle assembly and can directly interact with the meiotic cohesin REC8 and the kinetochore SGO1 (Shi et al. 2021). The rice PRD1 protein shows a dynamic localization on the whole chromosome during leptotene, transitioning to a discrete localization on centromeres after leptotene. The involvement of the PRD1 protein in spindle assembly seems variable between species, since it is not observed in maize (Wang et al. 2022b). In maize, PRD1 can also interact with PRD2, PRD3 and MTOPVIB but not directly with SPO11-1, SPO11-2, REC8 or SGO1 (Wang et al. 2022b) (Fig. 1). Whether or not these differences in the interaction network are conserved in other plant early recombinosomes will be important to understand in the future.

In budding yeast, Mer2 is phosphorylated by S phase Cdk to allow the association of the early recombinosome to the axial element (Panizza et al. 2011). Mer2 is also a coiled-coiled protein that connects the core complex with the axial element Hop1 and with Mre11 (Panizza et al. 2011; Rousová et al. 2021).

The Arabidopsis PRD3 (de Muyt et al. 2009; Lambing et al. 2023), its rice ortholog PAIR1 (Nonomura et al. 2004) and its maize orthologs (Wang et al. 2023a, b) are essential for DSB and bivalent formation*.* In Arabidopsis, PRD3 is not part of the RMM subcomplex as Mer2 but is essential to connect the core complex to factors of the MRN complex involved in the DSB resection (Vrielynck et al. 2021). In maize, PRD3 is also the main hub to connect the early recombinosome to the axial elements ASY1 and DSY2/ASY3 (Wang et al. 2023b) (Fig. 1). Whether or not PRD3 can be phosphorylated is still unknown, but PRD1 and PRD3 are the only components of the early recombinosome to be enriched in [S/T]Q sites (Lambing et al. 2023).

### PHS1, PRD2, and DFO, a divergent RMM subcomplex, connect to the axial elements of the synaptonemal complex (SC)

In *S. cerevisiae*, the RMM (Rec114, Mei4 and Mer2) proteins form an early recombinosome subcomplex important for association of the complex to DNA (reviewed in Yadav and Claeys Bouuaert 2021). The budding yeast RMM complex is composed of a Rec114 -Mei4 2:1 heterotrimer associated with a Mer2 homotetramer that can condense with DNA into reversible nucleoprotein clusters (Claeys Bouuaert et al. 2021a, b). In mammals REC114 interacts with the ankyrin repeat domain ANKRD31 that is specifically important for the regulation of DSB in the PAR region between X and Y sexual chromosomes (Boekhout et al. 2019; Acquaviva et al. 2020).

PHS1 is a divergent ortholog of the yeast Rec114 protein (Kumar et al. 2010), also poorly conserved in the mammalian REC114 (Kumar et al. 2018) and in DSB-1 from *C. elegans* (Hinman et al. 2021; Guo et al. 2022) (Supplementary Table 1). In plants, POOR HOMOLOGOUS SYNAPSIS (PHS1) was initially identified in maize (Pawlowski et al. 2004; Ronceret et al. 2009). It was originally considered that the signal of TUNEL assay (labelling DNA ends) observed in the maize *phs1* mutant meiocytes indicated that the formation of DSB was not impaired (Pawlowski et al. 2004), though early recombination was impaired based on drastic diminution of the RAD50 and RAD51 recombinases on chromatin (Pawlowski et al. 2004; Ronceret et al. 2009). As already described for *Zm spo11-1* mutants, we now think that the unexpected maize *phs1* phenotypes observed are due to residual Mu transposition creating few DSBs. In rice, a null *phs1* mutant forms only univalents (Yu et al. 2022). In bread wheat, PHS1 was immunolocalized on chromatin but did not colocalize with the meiotic axial element ASY1 (Khoo et al. 2012). However, in an Arabidopsis *phs1* null mutant created by CRISPR-Cas9 shows that PHS1 is dispensable for DSB formation even though it is still able to interact with MTOPVIB, PRD2 and DFO using Y2H assays as well as with ASY1 using BiFC assays (Vrielynck et al. 2021) (Fig. 1). Rec114 was recently involved in a mechanism boosting the formation of DSBs on the shorter budding yeast chromosomes in order to ensure their correct segregation via the obligate CO during meiosis (Murakami et al. 2020). The AlfaFold2 modeling prediction of the different PHS1 homolog structures, including the Arabidopsis and maize PHS1 proteins with intrinsically disordered regions, indicates that the Arabidopsis protein has lost part of an essential Pleckstrin-Homology domain (Daccache et al. 2023). It will be interesting to test if this phenomenon can explain why the role of PHS1 is not essential for DSB formation in Arabidopsis (Vrielynck et al. 2021), compared to rice (Yu et al. 2022) and maize (Pawlowski et al. 2004; Ronceret et al. 2009).

PRD2 is the divergent ortholog of Mei4 in *S. cerevisiae* (Maleki et al. 2007), rec24 in *S. pombe* (Bonfils et al. 2011) and MEI4 in mammals (Kumar et al. 2010, Acquaviva et al. 2020) (Supplementary Table 1). AtPRD2 (de Muyt et al. 2009) is also known as MULTIPOLAR SPINDLE1 (MPS1) for its role in spindle assembly (Jiang et al. 2009; Walker et al. 2018). AtPRD2 can auto-interact and can interact with AtSPO11-1, AtSPO11-2, AtMTOPVIB, AtPRD1, AtPHS1, AtDFO, and the axial elements AtASY1, AtASY3 and AtASY4 (Vrielynck et al. 2021) (Fig. 1). The function of PRD2 in DSB formation is conserved in rice (Wang et al. 2023a, b). In contrast to OsPRD1 (Shi et al. 2021) and AtPRD2/MPS1 (Jiang et al. 2009), OsPRD2 is not required for spindle assembly (Wang et al. 2023a, b).

Arabidopsis DSB Formation (DFO) was formally identified as a DSB factor essential for the initiation of recombination (Zhang et al. 2012) (Supplementary Table 1). A genetic formal functional characterization of the Arabidopsis *dfo* mutation was to check that it can eliminate the fragmentation phenotype of the *mre11* mutant in a *dfo/mre11* double mutant, the same as was observed for *spo11-1/mre11* double mutants (Puizina et al. 2004). AtDFO is a coiled-coil protein that can weakly interact with itself and interact with PHS1 and PRD2, SPO11-1 and MTOPVIB in yeast double hybrid experiments, as well as with ASY1 and ASY3 in BiFC assays (Vrielynck et al. 2021) (Fig. 1). DFO does not seem to have orthologs outside the plant kingdom and seems absent from bryophytes (Zhang et al. 2012; Thangavel et al. 2023). The DFO gene is duplicated in rice and maize and has not been analyzed in these species (Supplementary Table 1).

The RMM complex has a condensation activity that drives the assembly of the early recombinosome (Claeys Bouuaert et al. 2021b). In *S.cerevisiae*, Mei4 and Rec114 present DNA binding motifs with preference for branched DNA duplex structures while Mer2 has affinity for nucleosome (Rousová et al. 2021; Daccache et al. 2023).

Whether or not the regulation by the condensation mechanism of the early recombinosome co-expressed complex with DNA, as analyzed in budding yeast (Claeys Bouuaert et al. 2021a, b), is also valid for plant complex remains to be investigated.

## Regulation of the early recombination complex by the ATR/ATM kinase and heat stress

In budding yeast, Tel1/ATM and Mec1/ATR reduce DSBs to one DSB per quartet of chromatid (Zhang et al. 2011). Tel1 also reduces the number of DSB by phosphorylating Rec114 creating a negative loop of DSB formation (Carballo et al. 2013). In mice, the ATM/Tel1 homolog controls DSB formation (Lange et al. 2011), while the ATR/Mec1 homolog is required to complete meiotic recombination (Pacheco et al. 2018). In Arabidopsis, ATM is also essential for meiosis (Garcia et al. 2003). During Arabidopsis meiosis, ATM works with ATR (Culligan and Britt 2008). Both ATM and ATR protein kinases are needed to phosphorylate the DSB epigenetic marker γH2AX, labelling the nucleosomes of DNA extremities (Friesner et al. 2005; Vespa et al. 2007; Amiard et al. 2010) as well as several key meiotic proteins coordinating DSB repair and cell cycle checkpoint activation (Rotinger et al. 2015). It was proposed that ATM promotes RAD51 mediated meiotic DSB repair (Yao et al. 2020). In Arabidopsis, ATM not only signals the presence of a DSB by promoting recombinases in charge of homologous repair, it also affects the organization of chromatin loops and SC components (Kurzbauer et al. 2021). ATM is also required for genome stability at high 37–38 °C temperatures (Zhao et al. 2023), where the expression of ASY3, ASY4, RAD51 and DMC1 is downregulated while the expression of SPO11-1, PRD1, 2, and 3 is not impacted (Ning et al. 2021). At high temperatures, DSB are formed, but are not repaired to form CO, and are supposedly repaired using the sister chromatid instead of the homologous chromosome; a situation similar to what is observed in various *dmc1* mutants (Couteau et al. 1999, Wang et al. 2016; Colas et al. 2019; Szurman-Zubrzycka et al. 2019) as well as some *hop2* mutants (Uanschou et al. 2013). This meiotic DSB repair using the sister also requires RAD54, an essential RAD51 co-factor regulating recombinase activity (Hernandez Sanchez-Rebato et al. 2021). The effect of different temperatures on meiotic recombination affects the expression of several meiotic genes (Huang et al. 2021) and occurs at several levels (reviewed in Gutiérrez Pinzón et al. 2021; De Jaeger-Braet and Schnittger 2024).

In maize, the analysis of the role of ATM and ATR is complicated by the redundant duplication of both genes, but specific mutants show that while ATM does not trigger meiotic defect, ATR is essential for maternal fertility (Pedroza-Garcia et al. 2021) (Supplementary Table 1).

## Role of the chromosomal *axis* in the early recombinosome and the axial loop tethering model (ALTM)

In *S. cerevisiae* it was observed that while DSBs are mapped on chromatin loops, essential factors of the DSB machinery are associated with axial elements at the base of the loop (Blat et al. 2002). To reconcile these contradictory facts, the Axial-Loop Tethering Model (ALTM) suggests a change of conformation of the loop where DSBs are made and a tethering of the loop toward the axial element at the time of DSB formation (Blat et al. 2002; Pan et al. 2011; Panizza et al. 2011). The ALTM was also proposed to explain the formation of DSBs in *S. pombe* (Miyoshi et al. 2012).

In plants, the meiotic chromatin that is the substrate for DSB formation is also proposed to be organized with meiotic specific proteins associated at the basis of chromatin loops including the meiotic specific cohesin subunit REC8 (Lambing et al. 2020a, b). This expected organization in loop domains of the meiotic chromosomes was determined using nanoscopy (Kurzbauer et al. 2021). However fine scale plant meiotic chromosome organization has still not been validated by any conformation capture techniques and the potential Axial Loop Tethering at DSB remains hypothetical. However, the protein interaction network observed in Arabidopsis and maize between the early recombinosome, and the axial elements suggest such physical connections (Vrielynck et al. 2021; Wang et al. 2023a, b) (Fig. 1).

### ASY1, ASY3 and ASY4 are components of the plant meiotic axial elements

In Arabidopsis, several axial elements have been identified: ASY1 (Armstrong et al. 2002; Sanchez-Moran et al. 2007; Lambing et al. 2020a, b; Pochon et al. 2022), ASY3 (Ferdous et al. 2012) and ASY4 (Chambon et al. 2018). (Supplementary Table 1 and Fig. 1). ASY2 is an ASY1 homolog that does not have any known meiotic function (Armstrong et al. 2002) (Supplementary Table1). Other new members have been identified by proteomic interaction screen (Feng et al. 2023).

ASY1 is a HORMA domain protein, homolog with the mammals HORMAD1 and HORMAD2 (Wojtasz et al. 2009), forming the unsynapsed meiotic chromosomal axis (Armstrong et al. 2002; Lambing et al. 2020a, b) (Supplementary Table 1).

In Arabidopsis, ASY1, ASY3 or ASY4 are not required for the formation of most DSB, but are necessary for the HR repair choice, correct synapsis and formation of bivalents (Armstrong et al. 2002; Sanchez-Moran et al. 2007; Ferdous et al. 2012; Chambon et al. 2018). The mutant *asy1*, *asy3* mainly form univalents, while the mutant *asy4* only affects the formation of univalent in a few chromosomes (Chambon et al. 2018). In *asy3* mutants, the number of DSB and PRD3 foci are reduced suggesting a control of this axial element over DSB number (Ferdous et al. 2012, Lambing et al. 2023). Though AtASY1 is not necessary for DSB formation it controls global recombination landscape (Lambing et al. 2020a, b; Kuo et al. 2021). In rice, PAIR2 is the ortholog of ASY1 (Nonomura et al. 2006) (Supplementary Table 1). In contrast to Arabidopsis and rice, the maize ASY1/PAIR2 (Wang et al. 2023a, b) and the maize ASY3 named DESYNAPTIC 2 (DSY2), both localized on the axial elements (Lee et al. 2015) are also necessary for DSB formation in maize (Supplementary Table 1). ASY4 is duplicated in rice and maize and has not yet been characterized in these species (Supplementary Table 1).

AtASY1 is sequentially installed and removed from the chromosome by the action of the PCH2 (Lambing et al. 2015; Yang et al. 2020) and COMET (Balboni et al. 2020) proteins. In rice, the PCH2 homolog known as CRC1 (Miao et al. 2013) and the COMET homolog known as P31 or BVF1 (Ji et al. 2016; Zhou et al. 2017) are both necessary for DSB formation (Supplementary Table 1).

It therefore appears that the relative role of some axial element on the regulation of DSB formation could varies from species to species from indispensable to dispensable but conserving a global control on recombination landscape.

### The axial elements and the control of recombination

Another insightful approach for understanding the role of SC elements in recombination in plants has also come from the analysis of polyploid species. In tetraploid *Arabidopsis arenosa,* specific SC genes show adaptative signatures of natural selection, allowing correct recombination and segregation between four homologs instead of two (Lloyd and Bomblies 2016; Bomblies and Peichel 2022; Bomblies 2023). In *A. arenosa,* the meiotic cohesin REC8 (Morgan et al. 2022) and axial element proteins of the SC ASY1 and ASY3 are important for polyploid meiotic adaptation (Morgan et al. 2020). In autotetraploid *A. lyrata*, a variant of ASY3 is also associated with greater meiotic stability allowing polyploid viable transmission (Seers et al. 2020). It seems that a major issue for restricting pairwise partner connections in autopolyploids is solved by increasing CO interference (Morgan et al. 2021a, b). Considering the new models involving the SC and HEI10 coarsening proposed to explain CO interference (Morgan et al. 2021a, b; Durand et al. 2022; Morgan et al. 2024), it will be interesting to analyze what aspect of *A. arenosa* REC8, ASY1 and ASY3 variants affects SC structure and/or HEI10 coarsening to gain insights on the regulation of CO interference.

## A new model for the formation of DSB involving the tethering of both sister chromatids

**Fig. 2:**
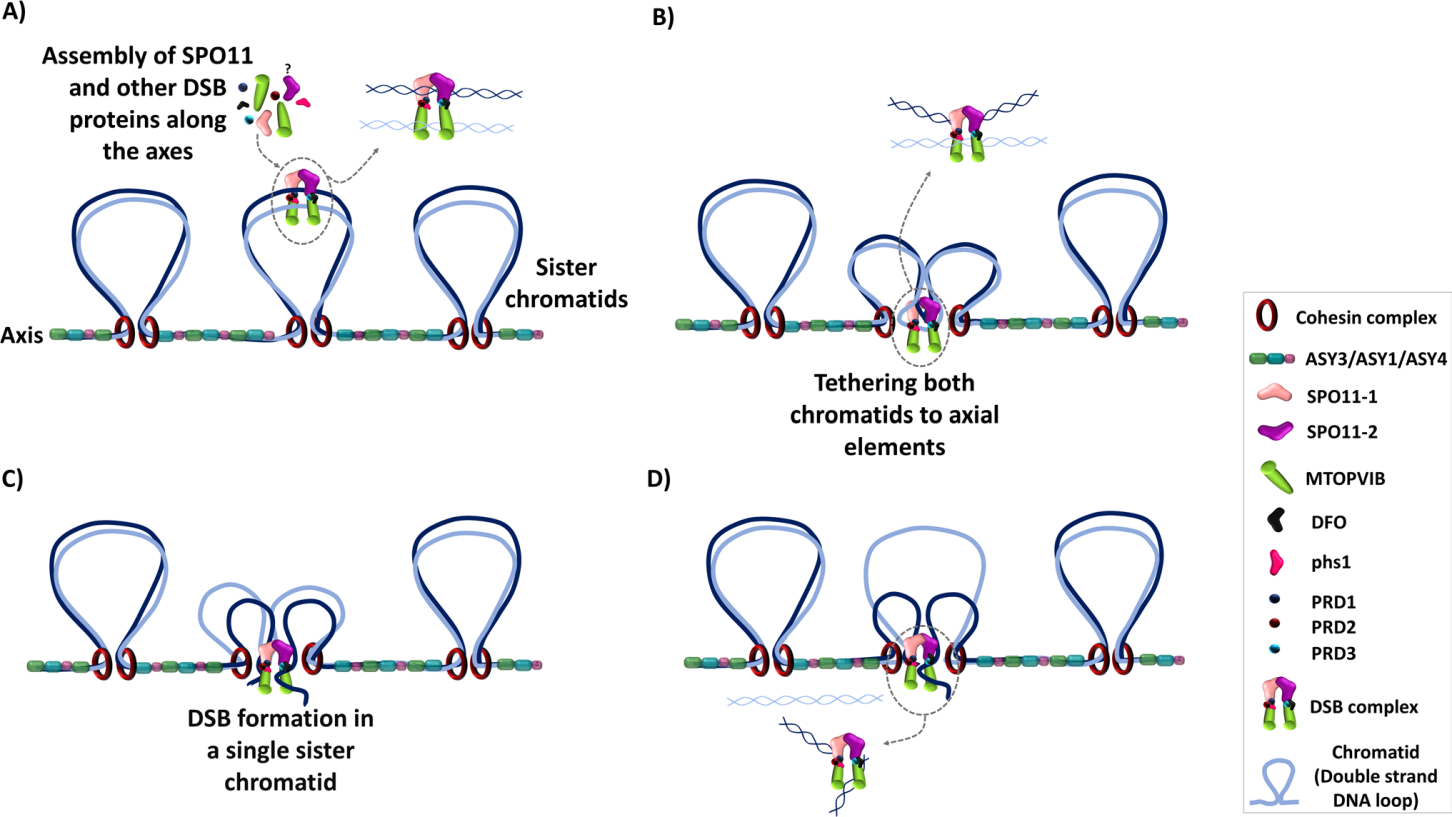
New model of DSB formation at unsynapsed chromosome axis involving the tethering of both sister chromatids to the axial elements and the formation of a DSB in only one of the sister chromatids (labelled in dark blue) by a ‘multi-key lock’ early recombinosome. The early recombinosome formed by three subcomplexes including the SPO11-1/2-MTOPVIB ‘core’ that conserves a topoisomerase VI-like structure. Two sister chromatids could be engaged inside the early recombinosome core complex (**A**) and concomitantly tethered toward axial elements **B**. Subsequently, double-strand break (DSB) occurs in only one of the sister chromatids, while the other remains intact **C** and **D**. The DSB forms in only one sister chromatid either by the ATLM or this new model, secures a rescue pathway of DSB repair by homologous recombination using the intact sister in case homologous chromosome cannot be found, or when DSBs are randomly affecting the same locus of both homologs. In the new model, the ATLM might still be necessary to direct the repair pathway toward homologous recombination instead of alternative NHEJ repair pathways

Based on theoretical assumptions of the DSB Repair model (Szostak et al. 1983), the repair machinery using the sister chromatid as an intact template to repair the homologous DNA with a double strand break can only work if the early recombinosome cuts only one sister chromatid at the same homologous position. In consequence, homologous repair between sisters is not supposed to work if both sisters are damaged at the same site. In accordance with this assumption, DSBs are experimentally found to occur only one per pair of chromatids in *S. cerevisiae* (Zhang et al. 2011). In maize, tetrad analysis of CO distribution also suggests that for each locus only one sister chromatid of each homolog is involved in the exchange to produce COs (Li et al. 2015) as was previously observed for the A1 hotspot (Yandeau-Nelson et al. 2006) and by classical genetical analysis (Rhoades 1932). Several figures showing the proposed ALTM consider the tethering of only one sister chromatid (Keeney et al. 2014; Grey and de Massy 2021, Yadav and Claeys Bouuaert 2021). In this hypothesis, the ALTM could explain how only one sister chromatid is broken at the basis of the loop. An alternative is that both sister chromatids are the substrate of the early recombinosome that would have the ability to cut in only one of them as they are engaged inside a topoisomerase-like complex (Claeys-Bouuaert et al. 2021). An argument in favor of this alternative model is that the ancestral conserved function of type VI topoisomerases is to resolve structural DNA entanglements between two adjacent DNA molecules engaged in the complex (Wendorff and Berger 2018, Yadav and Claeys Bouuaert 2021). Topoisomerase VI can create a transient breakage of only one of these two DNA molecules to allow the passage of the intact DNA molecule through the DSB that is finally religated (Wendorff and Berger 2018). Evolutionarily, compared to other topoisomerase VI enzymes, the early recombinosome core complex has conserved most of its general structure, as it is able to sense and exploit both DNA crossing and bends (Claeys Bouuaert et al. 2021a, b). On a theoretical basis, to create meiotic DSB, the early recombinosome core complex is expected to differ from other topoisomerase VI (and other type IIB topoisomerases in general) (Chang 2002) on the step of DSB religation (Robert et al. 2016a, b) and does not require the trans-passing of the intact DNA molecule through the transiently broken one. Whether or not the religation capacity and the gate trans passing of the early recombinosome core complex has been lost or is inhibited by some or the other sub-complexes is to our knowledge not yet understood. However, the lost ability of mTOPVIB and mTOPVIB-SPO11-1/2 to bind and hydrolyze ATP (Chen et al. 2024) suggests that these unnecessary functions were exapted for the purpose of the early recombinosome core-complex. Based on these considerations, we propose an alternative model in which the early recombinosome core-complex can recognize two sister chromatids in order to break only one of them at a particular genome site. This model is based on the ancestral recognition of topoisomerases type VI for two DNA molecules and expected conserved properties of the early recombinosome to engage two DNA molecules (Wang 2002; Claeys Bouuaert et al. 2021a, b). In this configuration, the observed gathering between the early recombinosome to the axial elements is not essential for the purpose of breaking only one chromatid if this role is already taken by the activity of the early recombinosome core-complex (Fig. 2). The ALTM could be involved in other processes such as the ability to connect the early recombinosome with the MRN machinery, as observed in Arabidopsis (Vrielynck et al. 2021), budding yeast (Rousová et al. 2021) and *C. elegans* (Girard et al. 2018). It could also make repair choice favoring Homologous Recombination (Vrielynck et al. 2021). Since plants do not require the MRN complex to form DSB (Supplementary Table 1), as it is the case in budding yeast or *C. elegans*, they might be good systems to analyze this new model possibility.

Interestingly it was recently found that loop extrusion also mediates DSB synapsis that are repaired by Non-Homologous End Joining (NHEJ) in somatic cells (Yang et al. 2023). It therefore seems that the meiotic regulation of the tethering loop observed in meiosis is a common feature for DSB repair by NHEJ and HR. In Arabidopsis, the 45S rDNA arrays of the nucleolar region are not repaired via HR but by NHEJ during meiosis and produce meiotic DSBs in a context that do not install ASY1 during leptotene and zygotene stages (Sims et al. 2019). This peculiar mode of meiotic recombination reinforces the idea that the axial elements of the SC are essential for the choice of the HR pathway.

## Conclusion and perspectives

The formation of DSBs during meiosis is due to the enzymatic activity of the early recombinosome complex, whose core subcomplex maintains the structure and characteristics of a topoisomerase type VI, associated with accessory factors that form two other subcomplexes. The absolute necessity of each of the multicomplex subunits can be seen as the first level for securing the formation of risky DSBs, as a multi-key lock system. With the absence of only one key/subunit the system is locked. This fact has direct implications for plant breeding strategies requiring plant DSB mutants for creating apomeiosis and apomixis in crops (Ronceret and Vielle-Calzada 2015; Underwood and Mercier 2022). It allows use of *SPO11-1* as well as all unique genes involved in DSB formation, such as *SPO11-2*, *MTOPVIB*, *PRD1*, *PRD2*, *PRD3* and *DFO,* alternatively in order to create, as in rice, useful plant meiotic DSB mutants, allowing apomeiosis (MiMe) (Fayos et al. 2019) and finally synthetic apomixis (Vernet et al. 2022). To make the same strategy in maize, we assume it will be important to select mutants coming from populations that do not have high Mutator activity. In maize, additional genes such as *DSY2/ASY3* and *PHS1* could also be used. In other species, the different necessity of these genes for DSB formation will require independent analyses for each plant of interest, since there is no phylogenetic prediction of their requirement for DSB formation and/or absence of somatic phenotype. The role and consequences of these differences is yet poorly understood as meiotic analysis are mainly realized in a handset of plant species (Arabidopsis, tomato, Brassica, rice, barley, maize and wheat).

The rapid divergence of some part of this early recombinosome system is intriguing (Vrielynck et al. 2021; Arter and Keeney 2024). Whether or not some subunits co-evolve and ensure DSB formation incompatibility between divergent species also remains to be investigated. It would help to understand the role of DSB formation in hybrid viability, speciation and polyploidization.

A more complete understanding of the structure-function of the different specificities of plant meiotic and somatic topoisomerase VI complexes would be interesting to investigate (Brinkmeier et al. 2022). The accessory factors BIN4 and RHL1 of the somatic TOPVI complex shows chromatin domain insulator-like function (Méteignier et al. 2022). Whether or not the accessory proteins of the early recombinosome have also peculiar chromatin domain binding specificities (such as nucleosome-free chromatin region preference) remains poorly explored in plants (Lambing et al. 2023).

The use of a multi-subunit topoisomerase enzyme globally ensures the formation of DSBs stochastically all along the chromosomes but only in the context of the meiotic-specific unsynapsed chromosome axis. This association probably occurs in order to couple the formation of DSBs with its subsequent processing via HR repair (Vrielynck et al. 2021). The core early recombinosome topoisomerase VI-like function suggests the essential possibility to act on both sister chromatids in order to break only one of them. The known small divergences in structure between archaeal topoisomerase VI and eukaryotic early recombinosome core complex (Claeys Bouuaert et al. 2021a, b) are compatible with this view. The fact that the core complex has lost its ability to bind and hydrolyze ATP also suggests this exaptation (Chen et al. 2024). Our proposed model of ‘one per pair of chromatids’ DSB formation by the early recombinosome core, ensures the theoretical possibility to use the unbroken sister as a backup intact matrix for HR, in case this DSB cannot be repaired using a homologous chromosome (Fig. 2). It also gives an alternative intact repair matrix in case random DSBs are made simultaneously on a similar locus on both homologs. To test whether the core early recombinosome can be associated with the two sister chromatids would require the difficult observation of the core complex on meiotic chromatin at the electron microscopy level. The continuous improvements in light nanoscopy resolution (Torres-García et al. 2022) might also give the possibility to test in vivo these difficult questions in the future. As both sister chromatids are almost indistinguishable, the HiC genomic techniques to differentiate these twins and prove this hypothesis in vivo are yet to be invented. New methods to analyze the mechanisms of the early recombinosome, based on the use of differential fluorescently-labelled DNA substrate and the use of FRET to observe possible association of two DNA molecules with the early recombinosome core complex, will be required to test the proposed ‘one per pair of chromatids’ DSB formation model.

### Author contribution statement

NR contributed to the manuscript and made the figures. AR wrote the manuscript and produced Supplementary Table 1.

## Supplementary Information

Below is the link to the electronic supplementary material.

Supplementary file1 (DOCX 90 kb)
