# Supplementary material for: The plant early recombinosome: a high security complex to break DNA during meiosis
Source: Plant Reprod. 2024 Sep 27;37(4):421–40. doi: 10.1007/s00497-024-00509-7 (PMC11511760; doi:10.1007/s00497-024-00509-7)
Supplement: Supplementary file 1 — Supplementary file1 (DOCX 90 kb) [file 497_2024_509_MOESM1_ESM.docx]

| ***S. cerevisiae*** | ***S. pombe*** | ***A. thaliana*** | ***O. sativa*** | ***Z. mays*** | ***M. musculus*** |
| --- | --- | --- | --- | --- | --- |
| **SPO11 core complex** | | | | | |
| **Spo11**  (Keeney S., et al.1997;  Bergerat A. et al. 1997 ;  Jonhson D. et al. 2021) | **Rec12**  (Lin Y. and Smith G. R. 1994 ;  Kan F. et al. 2011 ;  Miyoshi T. et al. 2012 ; Hou H. et al. 2021) | **SPO11-1**  (Hartung F. and Puchta H. 2000 ; Grelon M. et al. 2001 ; Stacey N. J. et al. 2006 ; Lambing C. et al. 2022) | **SPO11-1**  (Jain M et al 2006 ;  Yu H. et al. 2010) | **SPO11-1 (𝛂 and 𝛃)**  (Ku J-C. et al. 2020) | **SPO11 (𝛂 and 𝛃)**  (Romanienko P. J. and Camerini-Otero R. D. 2000 ; Baudat F. et al. 2000 ;  Kauppi L. et al. 2011) |
|  |  | **SPO11-2**  (Hartung F. and Puchta H. 2000 ; Grelon M. et al. 2001 ; Stacey N. J. et al. 2006) | **SPO11-2**  (Jain M. et al 2006 ; Fayos I. et al. 2020) | **SPO11-2**  (Li M. et al. 2022) |  |
|  |  | SPO11-3  (Yin Y. et al. 2002) | SPO11-3  (Jain M. 2006) | SPO11-3 |  |
|  |  |  | **SPO11-4**  (An XJ. et al. 2011 ; Fayos I. et al. 2020)  SPO11-5  (An XJ. et al. 2011) |  |  |
| **Rec102**  (Malone R. E. et al. 1991 ; Bhargava J. et al. 1992 ; Galbraith A. M. and Malone R. E. 1992 ;  Claeys Bouuaert C. et al. 2021)  **Rec104**  (Malone R. E. et al. 1991 ; Bhargava J. et al. 1992 ; Galbraith A. M. and Malone R. E. 1992 ;  Claeys Bouuaert C. et al. 2021) | **Rec6**  (Lin Y. and Smith G.R. 1994 ; Cervantes M. D. et al. 2000 ; Miyoshi T. et al. 2012) | **MTOPOVIB**  (Vrielynck N. et al. 2016 ; Tang Y. et al 2017 ;  Chen H-W. et al. 2024) | **MTOPVIB**  (Xue Z. et al. 2016 ; Fu M. et al. 2016 ; Xue Z. et al. 2019) | **MTOPVIB**  (Jing JL. et al. 2020) | **TOPVIBL**  (Robert T. et al. 2016 ;  Nore A. et al. 2022) |
| **Ski8/Rec103**  (Gardiner J. M. et al. 1997 ; Arora et al. 2004 ;  Claeys Bouuaert C. et al. 2021) | **Rec14**  (Evans D. H. et al. 1997 ; Steiner S. et al. 2010 ; Miyoshi T. et al. 2012) | SKI8/VIP3  (Jolivet S. et al. 2006 ; Dorcey E. et al. 2012) | SKI8 | SKI8 | WDR61  (Hou Y. et al. 2024) |
| **RMM complex** | | | | | |
|  |  | **PRD1**  (De Muyt A. et al. 2007 ;  Tang, Y. et al. 2017) | **PRD1**  (Shi W. et al. 2021) | **PRD1**  (Wang Y. et al. 2022) | **MEI1**  (Libby B. J. et al. 2003) |
|  |  |  |  |  | **ANKRD31**  (Boekhout M. et al. 2019 ;  Papanikos F. et al. 2019  Xu J. et al. 2023) |
| **Rec114**  (Menees T. M. and Roeder G. S. 1989 ;  Carballo J.A. et al. 2013;  Murakami H. et al 2020) | **Rec7**  (Molnar M. et al. 2001 ; Steiner S. et al. 2010) | **PHS1**  (Vrielynck, N. et al. 2021) | **PHS1**  (Yu H. et al. 2022) | **PHS1**  (Pawlowski W.P. et al. 2004 ; Ronceret et al. 2009) | **REC114**  (Kumar R. et al. 2018  Nore T. et al. 2022) |
| **Mei4**  (Menees T. M. and Roeder G. S. 1989  Claeys Bouuaert C. et al. 2021) | **Rec24**  (Bonfils S. et al. 2011) | **PRD2**  (De Muyt A. et al. 2009 ;  Walker et al. 2018) | **PRD2**  (Wang C., et al. 2023) | PRD2 | **MEI4**  (Kumar R. et al. 2010  Kumar R. et al. 2015) |
| **Mer2**  (Engebrecht J. et al. 1990 ;  Daccache D. et al. 2023) | **Rec15**  (Lin Y. and Smith G. R. 1995 ; Miyoshi T. et al. 2012) | **PRD3**  (De Muyt A. et al. 2009 ;  Lambing C. et al. 2022) | **PAIR1**  (Nonomura K.I. et al. 2004) | **PRD3**  (Wang Y. et al. 2023) | **IHO1**  (Stanzione M. et al. 2016 ;  Laroussi H. et al. 2023 ; Dereli I. et al. 2024) |
|  |  | **DFO**  (Zhang, C. et al. 2012) | DFO1  DFO2 | DFO1  DFO2 |  |
| **MRX complex** | | | | | |
| Mre11  (Johzuka K. and Ogawa H. 1995) | Rad32  (Tavassoli M. et al. 1995 ; Young J. A. et al. 2004 ; Hartsuiker E. et al. 2009) | MRE11  (Gallego M. E. et al. 2001 ; Puzina J. et al. 2004 ; Bleuyard J. Y., et al. 2004 ; Waterworth W. M. et al. 2007) | MRE11  (Ji J. et al. 2013) | MRE11A  MRE11B  (Waterworth WM. et al. 2007) | MRE11  (Cherry S. M. et al. 2007 ; Zhang B., 2020) |
| Rad50  (Game J. C. et al. 1980 ;  Alani E. et al. 1990 ;  Johzuka K. and Ogawa H. 1995) | Rad50  (Young, J. A. et al. 2004) | RAD50  (Gallego M. E. et al. 2001 ; Bleuyard J. Y. et al. 2004 ; Waterworth W. M. et al. 2007) | RAD50 | RAD50  (Waterworth WM. et al. 2007) | RAD50  (Cherry S. M. et al. 2007 ; Zhang B. 2020 ;  Liu Y. et al. 2024) |
| Xrs2  (Ivanov E. L. et al. 1992) | Nbs1  (Farah J. A. et al. 2005) | NBS1  (Gallego M. E. et al. 2001 ; Bleuyard J. Y. et al. 2004 ; Waterworth W. M. et al. 2007) | NBS1 | NBS1  (Waterworth WM. et al. 2007) | NBS1  (Cherry S. M. et al. 2007 ; Zhang B. et al. 2020) |
| **ATM/ATR kinases** | | | | | |
| Tel1 | Rad3 | ATM | ATM-A | ATM-A | ATM |
| (Usui T. et al. 2001,  Carballo J.A. et al. 2008,  Zhang L. et al. 2011) | (Jimenez G. et al. 1992 ; Bentley N.J. et al. 1996) | (Culligan KM. and Britt AB. 2008 ; Kurzbauer M-T. et al. 2020 ; Zhao J. et al. 2023) | ATM-B  (Zhang C. et al. 2020) | ATM-B  (Pedroza-Garcia J.A. et al. 2021) | (Xu Y. et al. 1996 ;  Lange J. et al. 2011) |
| Mec1  (Zhang L. et al 2011 ;  Gray S. et al. 2013) |  | ATR  (Culligan KM. and Britt A.B. 2008) | ATR-A  ATR-B | ATR-A  ATR-B  (Pedroza-Garcia J.A. et al. 2021) | ATR  (Keegan et al. 1996 ;  Widger A. et al. 2018 ;  Pacheco S. et al. 2018) |
| **Chromosomal axial elements** | | | | | |
| **Hop1**  (Hollingsworth N. M. and Byers B. 1989 ;  Carballo et al. 2008 ;  Milano C.R. et al. 2024) |  | **ASY1**  (Armstrong S. et al. 2002 ;  Sanchez-Moran E. et al. 2007 ; Lambing C. et al. 2020 ; Pochon G. et al. 2022 ; Feng C. et al. 2023)  ASY2  (Caryl A. P. et al. 2000) | **PAIR2**  (Nonomura K.I., et al. 2004 ;  (Nonomura K.I., et al. 2006) | **ASY1**  (Wang Y., et al. 2023) | **HORMAD1**  **HORMAD2**  (Wojtasz L. et al 2009 ; Fukuda, T. et al. 2010 ; Shin Y. H. et al. 2010) |
| **Red1**  (Thompson E. A. and Roeder G. S. 1989 ; Rockmill B. and Roeder G. S. 1990 ; Blat Y. et al. 2002 ; Carballo J.A. et al. 2008) |  | **ASY3**  (Ferdous M. et al. 2012 ;  Feng C. et al. 2023) | **PAIR3**  (Yuan W. et al. 2009 ; Wang K. et al. 2011) | **DSY2**  (Franklin A.E. et al. 2003 ; Lee D.H. et al. 2015) | **SYCP2**  (Kolas N.K. et al. 2004 ;  Kouznetsova A. et al. 2006) |
|  |  | **ASY4**  (Chambon A. et al. 2018) | ASY4-A  ASY4-B | ASY4-A  ASY4-B | **SYCP3**  (Kolas N.K. et al. 2004 ;  Kouznetsova A. et al 2006) |
| Pch2  (San-Segundo P.A and Roeder G.S. 1999 ; Ho H.C. and Burgess S.M. 2011 ;  Chen C. et al. 2014) |  | **PCH2**  (Lambing C. et al. 2015 ; Yang C. et al. 2020) | **CRC1**  (Miao C. et al. 2013) | PCH2 | **TRIP13/PCH2**  (Li X.C. and Schimenti J.C. 2007 ; Wojtasz, L. et al 2009 ;  Roig I. et al. 2010) |
| Tyc1  (Schuyler S.C. et al. 2018) |  | **P31/COMET**  (Balboni M. et al. 2020) | **P31 / BVF1**  (Ji J. et al. 2016 ;  Zhou L. et al. 2017) | P31 | P31 |

**Table 1.** List of indispensable meiotic DSB formation proteins in different model species.

Proteins with conserved sequence and /or demonstrated structure/function between species are on a same line, when a protein is part of a family, the members involved in meiotic DSB formation are in red and in green when not indispensable for DSB formation, in blue when they have no meiotic function, in black when its role in DSB formation is not known. The protein in bold have an expression enriched during meiosis.

References for Table 1:

Alani E., Padmore R., Kleckner N. 1990. Analysis of wild-type and rad50 mutants of yeast suggests an intimate relationship between meiotic chromosome synapsis and recombination. ***Cell.*** 61(3):419-36. doi: 10.1016/0092-8674(90)90524-i. PMID: 2185891.

An X.J., Deng Z.Y., Wang T. 2011. OsSpo11-4, a Rice Homologue of the Archaeal TopVIA Protein, Mediates Double-Strand DNA Cleavage and Interacts with OsTopVIB. ***PLoS ONE*** 6(5): e20327. doi:10.1371/journal.pone.0020327

Armstrong S.J., Caryl A.P., Jones G.H., Franklin F.C. 2002. Asy1, a protein required for meiotic chromosome synapsis, localizes to axis-associated chromatin in Arabidopsis and Brassica. ***J Cell Sci.*** 115(Pt 18):3645-55. doi: 10.1242/jcs.00048. PMID: 12186950.

Arora C., Kee K., Maleki S., Keeney S. 2008. Antiviral protein Ski8 is a direct partner of Spo11 in meiotic DNA break formation, independent of its cytoplasmic role in RNA metabolism. ***Mol Cell.*** 13(4):549-59. doi: 10.1016/s1097-2765(04)00063-2. PMID: 14992724.

Balboni M., Yang C., Komaki S., Brun J., Schnittger A. 2020. COMET Functions as a PCH2 Cofactor in Regulating the HORMA Domain Protein ASY1. ***Curr Biol.*** 30(21):4113-4127.e6. doi: 10.1016/j.cub.2020.07.089. Epub 2020 Aug 27. PMID: 32857973.

Baudat, F., Manova, K., Yuen, J. P., Jasin, M. & Keeney, S. 2000. Chromosome synapsis defects and sexually dimorphic meiotic progression in mice lacking Spo11. ***Mol. Cell*** 6, 989–998.

Bentley NJ., Holtzman DA., Flaggs G., Keegan KS., DeMaggio A., Ford JC., Hoekstra M., Carr AM. 1996. The *Schizosaccharomyces pombe* rad3 checkpoint gene. ***EMBO J.*** 15(23):6641-51. PMID: 8978690; PMCID: PMC452488.

Bergerat A., de Massy B., Gadelle D., Varoutas P.C., Nicolas A., Forterre P. 1997. An atypical topoisomerase II from Archaea with implications for meiotic recombination. ***Nature.*** 386(6623):414-7. doi: 10.1038/386414a0. PMID: 9121560.

Bhargava, J., Engebrecht, J., Roeder, G. S. 1992. The rec102 mutant of yeast is defective in meiotic recombination and chromosome synapsis. ***Genetics*** 130, 59–69.

Blat Y., Protacio R.U., Hunter N., Kleckner N. 2002. Physical and functional interactions among basic chromosome organizational features govern early steps of meiotic chiasma formation. ***Cell.*** 111(6):791-802. doi: 10.1016/s0092-8674(02)01167-4. PMID: 12526806.

Bleuyard, J. Y., Gallego, M. E., White, C. I. 2004. Meiotic defects in the Arabidopsis rad50 mutant point to conservation of the MRX complex function in early stages of meiotic recombination. ***Chromosoma*** 113, 197–203.

Boekhout M., Karasu ME., Wang J., Acquaviva L., Pratto F., Brick K., Eng DY., Xu J, Camerini-Otero RD., Patel DJ., Keeney S. 2019. REC114 Partner ANKRD31 Controls Number, Timing, and Location of Meiotic DNA Breaks. ***Mol Cell.*** 74(5):1053-1068.e8. doi: 10.1016/j.molcel.2019.03.023. Epub 2019 Apr 16. PMID: 31003867; PMCID: PMC6555648.

Bonfils, S., Rozalen, A. E., Smith, G. R., Moreno, S., Martin-Castellanos, C. 2011. Functional interactions of Rec24, the fission yeast ortholog of mouse Mei4, with the meiotic recombination-initiation complex. ***J. Cell Sci.*** 124, 1328–1338.

Carballo J.A., Johnson A.L., Sedgwick S.G., Cha R.S. 2008. Phosphorylation of the axial element protein Hop1 by Mec1/Tel1 ensures meiotic interhomolog recombination. ***Cell.*** 132(5):758-70. doi: 10.1016/j.cell.2008.01.035. PMID: 18329363.

Carballo JA., Panizza S., Serrentino ME., Johnson AL., Geymonat M., Borde V., Klein F., Cha RS. 2013. Budding yeast ATM/ATR control meiotic double-strand break (DSB) levels by down-regulating Rec114, an essential component of the DSB-machinery. ***PLoS Genet.*** 9(6):e1003545. doi: 10.1371/journal.pgen.1003545. Epub 2013 Jun 27. PMID: 23825959; PMCID: PMC3694840.

Caryl, A. P., Armstrong, S. J., Jones, G. H., Franklin, F. C. 2000. A homologue of the yeast HOP1 gene is inactivated in the Arabidopsis meiotic mutant *asy1*. ***Chromosoma*** 109, 62–71.

Cervantes, M. D., Farah, J. A., Smith, G. R. 2000. Meiotic DNA breaks associated with recombination in *S. pombe*. ***Mol. Cell*** 5, 883–888.

Chambon A., West A., Vezon D., Horlow C., De Muyt A., Chelysheva L., Ronceret A., Darbyshire A., Osman K., Heckmann S., Franklin F.C.H., Grelon M. 2018. Identification of ASYNAPTIC4, a Component of the Meiotic Chromosome Axis. ***Plant Physiol.*** 178(1):233-246. doi: 10.1104/pp.17.01725. Epub 2018 Jul 12. PMID: 30002256; PMCID: PMC6130017.

Chen C., Jomaa A., Ortega J., Alani EE. 2014. Pch2 is a hexameric ring ATPase that remodels the chromosome axis protein Hop1. ***Proc Natl Acad Sci U S A.*** 111(1):E44-53. doi: 10.1073/pnas.1310755111. Epub 2013 Dec 23. PMID: 24367111; PMCID: PMC3890899.

Chen HW., Yeh HY., Chang CC., Kuo WC., Lin SW., Vrielynck N., Grelon M., Chan NL., Chi P. 2024. Biochemical characterization of the meiosis-essential yet evolutionarily divergent topoisomerase VIB-like protein MTOPVIB from *Arabidopsis thaliana*. ***Nucleic Acids Res.*** 52(8):4541-4555. doi: 10.1093/nar/gkae181. PMID: 38499490; PMCID: PMC11077084.

Cherry S.M., Adelman C.A., Theunissen J.W., Hassold T.J., Hunt P.A., Petrini J.H. 2007. The Mre11 complex influences DNA repair, synapsis, and crossing over in murine meiosis. ***Curr Biol.*** 17(4):373-8. doi: 10.1016/j.cub.2006.12.048. Epub 2007 Feb 8. PMID: 17291760; PMCID: PMC1839861.

Claeys Bouuaert C., Tischfield S.E., Pu S., Mimitou E.P., Arias-Palomo E., Berger J.M., Keeney S. 2021. Structural and functional characterization of the Spo11 core complex. ***Nat Struct Mol Biol.*** 28(1):92-102. doi: 10.1038/s41594-020-00534-w. Epub 2021 Jan 4. PMID: 33398171; PMCID: PMC7855791.

Culligan KM, Britt AB. 2008. Both ATM and ATR promote the efficient and accurate processing of programmed meiotic double-strand breaks. ***Plant J.*** 55(4):629-38. doi: 10.1111/j.1365-313X.2008.03530.x. Epub 2008 Apr 24. PMID: 18435824.

Daccache D., De Jonge E., Liloku P., Mechleb K., Haddad M., Corthaut S., Sterckx YG., Volkov AN., Claeys Bouuaert C. 2023. Evolutionary conservation of the structure and function of meiotic Rec114-Mei4 and Mer2 complexes. ***Genes Dev.*** 37(11-12):535-553. doi: 10.1101/gad.350462.123. Epub 2023 Jul 13. PMID: 37442581; PMCID: PMC10393190.

De Muyt A., Vezon D., Gendrot G., Gallois J.L., Stevens R., Grelon M. 2007. AtPRD1 is required for meiotic double strand break formation in *Arabidopsis thaliana*. ***EMBO J.*** 26(18):4126-37. doi: 10.1038/sj.emboj.7601815. Epub 2007 Aug 30. PMID: 17762870; PMCID: PMC2230667.

De Muyt A., Pereira L., Vezon D., Chelysheva L., Gendrot G., Chambon A., Lainé-Choinard S., Pelletier G., Mercier R., Nogué F., Grelon M. 2009. A high throughput genetic screen identifies new early meiotic recombination functions in *Arabidopsis thaliana.* ***PLoS Genet.*** 2009 Sep;5(9):e1000654. doi: 10.1371/journal.pgen.1000654. Epub 2009 Sep 18. PMID: 19763177; PMCID: PMC2735182.

Dereli I., Telychko V., Papanikos F., Raveendran K., Xu J., Boekhout M., Stanzione M., Neuditschko B., Imjeti NS, Selezneva E., Tuncay H., Demir S., Giannattasio T., Gentzel M., Bondarieva A., Stevense M., Barchi M., Schnittger A., Weir JR., Herzog F., Keeney S., Tóth A. 2024. Seeding the meiotic DNA break machinery and initiating recombination on chromosome axes. ***Nat Commun.*** 15(1):2941. doi: 10.1038/s41467-024-47020-1. PMID: 38580643; PMCID: PMC10997794.

Dorcey E., Rodriguez-Villalon A., Salinas P., Santuari L., Pradervand S., Harshman K., Hardtke CS. 2012. Context-dependent dual role of SKI8 homologs in mRNA synthesis and turnover. ***PLoS Genet.***8(4):e1002652. doi: 10.1371/journal.pgen.1002652. Epub 2012 Apr 12. PMID: 22511887; PMCID: PMC3325215.

Engebrecht, J., Hirsch, J., Roeder, G. S. 1990. Meiotic gene conversion and crossing over: their relationship to each other and to chromosome synapsis and segregation. ***Cell*** 62, 927–937.

Evans, D. H., Li, Y. F., Fox, M. E., Smith, G. R. 1997. A WD repeat protein, Rec14, essential for meiotic recombination in *Schizosaccharomyces pombe*. ***Genetics*** 146, 1253–1264.

Farah JA., Cromie G., Steiner WW., Smith GR. 2005. A novel recombination pathway initiated by the Mre11/Rad50/Nbs1 complex eliminates palindromes during meiosis in *Schizosaccharomyces pombe*. ***Genetics.*** 169(3):1261-74. doi: 10.1534/genetics.104.037515. Epub 2005 Jan 16. PMID: 15654094; PMCID: PMC1449568.

Fayos I., Meunier AC., Vernet A., Navarro-Sanz S., Portefaix M., Lartaud M., Bastianelli G., Périn C., Nicolas A., Guiderdoni E. 2020. Assessment of the roles of SPO11-2 and SPO11-4 in meiosis in rice using CRISPR/Cas9 mutagenesis. ***J Exp Bot.*** 71(22):7046-7058. doi: 10.1093/jxb/eraa391. PMID: 32842152.

Ferdous M., Higgins J.D., Osman K., Lambing C., Roitinger E., Mechtler K., Armstrong S.J., Perry R., Pradillo M., Cuñado N., Franklin FC. 2012. Inter-homolog crossing-over and synapsis in Arabidopsis meiosis are dependent on the chromosome axis protein AtASY3. ***PLoS Genet.*** 8(2):e1002507. doi: 10.1371/journal.pgen.1002507. Epub 2012 Feb 2. PMID: 22319460; PMCID: PMC3271061.

Franklin A.E., Golubovskaya I.N.., Bass H.W., Cande W.Z. 2003. Improper chromosome synapsis is associated with elongated RAD51 structures in the maize *desynaptic2* mutant. ***Chromosoma.*** 112(1):17-25. doi: 10.1007/s00412-003-0242-8. Epub 2003 Jun 14. PMID: 12811575.

Fu M., Wang C., Xue F., Higgins J., Chen M., Zhang D., Liang W. 2016. The DNA Topoisomerase VI-B Subunit OsMTOPVIB Is Essential for Meiotic Recombination Initiation in Rice. ***Mol Plant.*** 9(11):1539-1541. doi: 10.1016/j.molp.2016.07.006. Epub 2016 Jul 28. PMID: 27477684.

Fukuda, T., Daniel, K., Wojtasz, L., Toth, A. & Hoog, C. 2010. A novel mammalian HORMA domain-containing protein, HORMAD1, preferentially associates with unsynapsed meiotic chromosomes. ***Exp. Cell Res.*** 316, 158–171.

Galbraith, A. M., Malone, R. E. 1992. Characterization of REC104, a gene required for early meiotic recombination in the yeast Saccharomyces cerevisiae. ***Dev. Genet.*** 13, 392–402.

Gallego M.E., Jeanneau M., Granier F., Bouchez D., Bechtold N., White C.I. 2001. Disruption of the Arabidopsis RAD50 gene leads to plant sterility and MMS sensitivity. ***Plant J.*** 25(1):31-41. doi: 10.1046/j.1365-313x.2001.00928.x. PMID: 11169180.

Game, J. C., Zamb, T. J., Braun, R. J., Resnick, M. & Roth, R. M. 1980. The role of radiation (rad) genes in meiotic recombination in yeast. ***Genetics*** 94, 51–68.

Gardiner, J. M., Bullard, S. A., Chrome, C. & Malone, R. E. 1997. Molecular and genetic analysis of REC103, an early meiotic recombination gene in yeast. ***Genetics*** 146, 1265–1274.

Gray S., Allison R.M., Garcia V., Goldman A.S., Neale MJ. 2013. Positive regulation of meiotic DNA double-strand break formation by activation of the DNA damage checkpoint kinase Mec1(ATR). ***Open Biol.*** 3(7):130019. doi: 10.1098/rsob.130019. PMID: 23902647; PMCID: PMC3728922.

Grelon M., Vezon D., Gendrot G., Pelletier G. 2001. AtSPO11-1 is necessary for efficient meiotic recombination in plants. ***EMBO J.*** 20(3):589-600. doi: 10.1093/emboj/20.3.589. PMID: 11157765; PMCID: PMC133473.

Hartsuiker E., Mizuno K., Molnar M., Kohli J., Ohta K., Carr A.M. 2009. Ctp1^CtIP^ and Rad32^Mre11^ nuclease activity are required for Rec12^Spo11^ removal, but Rec12^Spo11^ removal is dispensable for other MRN-dependent meiotic functions. ***Mol Cell Biol.*** 2009 Apr;29(7):1671-81. doi: 10.1128/MCB.01182-08. Epub 2009 Jan 12. PMID: 19139281; PMCID: PMC2655602.

Hartung, F., Puchta, H. 2000. Molecular characterisation of two paralogous SPO11 homologues in Arabidopsis thaliana. ***Nucleic Acids Res.*** 28, 1548–1554 (2000).

Hartung F., Wurz-Wildersinn R., Fuchs J., Schubert I., Suer S., Puchta H. 2007. The catalytically active tyrosine residues of both SPO11-1 and SPO11-2 are required for meiotic double-strand break induction in Arabidopsis. ***Plant Cell.*** 19(10):3090-9. doi: 10.1105/tpc.107.054817. Epub 2007 Oct 26. PMID: 17965269; PMCID: PMC2174718.

Ho H.C., Burgess S.M. 2011. Pch2 acts through Xrs2 and Tel1/ATM to modulate interhomolog bias and checkpoint function during meiosis. ***PLoS Genet.*** 7(11):e1002351. doi: 10.1371/journal.pgen.1002351. Epub 2011 Nov 3. PMID: 22072981; PMCID: PMC3207854.

Hollingsworth, N. M., Byers, B. 1989. HOP1: a yeast meiotic pairing gene. ***Genetics*** 121, 445–462.

Hou H., Kyriacou E., Thadani R., Klutstein M., Chapman JH., Cooper J.P. 2021. Centromeres are dismantled by foundational meiotic proteins Spo11 and Rec8. ***Nature.*** 591(7851):671-676. doi: 10.1038/s41586-021-03279-8. Epub 2021 Mar 3. PMID: 33658710; PMCID: PMC8843027.

Hou Y., Zhang C., Liu L., Yu Y., Shi L., Qin Y. 2024. WDR61 ablation triggers R-loop accumulation and suppresses breast cancer progression. ***FEBS J.*** doi: 10.1111/febs.17145. Epub ahead of print. PMID: 38708718.

Ivanov, E. L., Korolev, V. G. & Fabre, F. 1992. XRS2, a DNA repair gene of *Saccharomyces cerevisiae*, is needed for meiotic recombination. ***Genetics*** 132, 651–664.

Jain M., Tyagi AK., Khurana J.P. 2006. Overexpression of putative topoisomerase 6 genes from rice confers stress tolerance in transgenic Arabidopsis plants. ***FEBS J.*** 273(23):5245-60. doi: 10.1111/j.1742-4658.2006.05518.x. PMID: 17116242.

Ji J., Tang D., Wang M., Li Y., Zhang L., Wang K., Li M, Cheng Z. 2013. MRE11 is required for homologous synapsis and DSB processing in rice meiosis. ***Chromosoma.*** 122(5):363-76. doi: 10.1007/s00412-013-0421-1. Epub 2013 Jun 22. PMID: 23793712.

Ji J., Tang D., Shen Y., Xue Z., Wang H., Shi W., Zhang C., Du G., Li Y., Cheng Z. 2016. P31comet, a member of the synaptonemal complex, participates in meiotic DSB formation in rice. ***Proc Natl Acad Sci U S A*** 113(38):10577-82. doi: 10.1073/pnas.1607334113. Epub 2016 Sep 6. PMID: 27601671; PMCID: PMC5035842.

Jimenez G., Yucel J., Rowley R., Subramani S. 1992. The rad3+ gene of *Schizosaccharomyces pombe* is involved in multiple checkpoint functions and in DNA repair. ***Proc Natl Acad Sci U S A.*** 89(11):4952-6. doi: 10.1073/pnas.89.11.4952. PMID: 1594599; PMCID: PMC49206.

Jing JL., Zhang T., Kao YH., Huang TH.., Wang CR., He Y. 2020. ZmMTOPVIB Enables DNA Double-Strand Break Formation and Bipolar Spindle Assembly during Maize Meiosis. ***Plant Physiol.*** 184(4):1811-1822. doi: 10.1104/pp.20.00933. Epub 2020 Oct 19. PMID: 33077613; PMCID: PMC7723106.

Johzuka, K. & Ogawa, H. 1995. Interaction of Mre11 and Rad50: two proteins required for DNA repair and meiosis-specific double-strand break formation in *Saccharomyces cerevisiae*. ***Genetics*** 139, 1521–1532.

Johnson D., Crawford M., Cooper T., Claeys Bouuaert C., Keeney S., Llorente B., Garcia V., Neale MJ. 2021. Concerted cutting by Spo11 illuminates meiotic DNA break mechanics. ***Nature.*** 594(7864):572-576. doi: 10.1038/s41586-021-03389-3. Epub 2021 Jun 9. PMID: 34108687; PMCID: PMC7611867.

Kan F., Davidson MK., Wahls WP. 2011. Meiotic recombination protein Rec12: functional conservation, crossover homeostasis and early crossover/non-crossover decision. ***Nucleic Acids Res.*** 39(4):1460-72. doi: 10.1093/nar/gkq993. Epub 2010 Oct 28. PMID: 21030440; PMCID: PMC3045620.

Kauppi L., Barchi M., Baudat F., Romanienko PJ., Keeney S., Jasin M. 2011. Distinct properties of the XY pseudoautosomal region crucial for male meiosis***. Science.*** 331(6019):916-20. doi: 10.1126/science.1195774. PMID: 21330546; PMCID: PMC3151169.

Keegan KS., Holtzman DA., Plug AW., Christenson ER., Brainerd EE., Flaggs G., Bentley NJ., Taylor EM., Meyn MS., Moss SB., Carr AM., Ashley T., Hoekstra MF. 1996. The Atr and Atm protein kinases associate with different sites along meiotically pairing chromosomes. ***Genes Dev.***10(19):2423-37. doi: 10.1101/gad.10.19.2423. PMID: 8843195.

Keeney, S., Giroux, C. N. & Kleckner, N. 1997. Meiosis-specific DNA double-strand breaks are catalyzed by Spo11, a member of a widely conserved protein family. ***Cell*** 88, 375–384.

Kolas N.K., Yuan L., Hoog C., Heng H.H., Marcon E., Moens P.B. 2004. Male mouse meiotic chromosome cores deficient in structural proteins SYCP3 and SYCP2 align by homology but fail to synapse and have possible impaired specificity of chromatin loop attachment. ***Cytogenet Genome Res.*** 105(2-4):182-8. doi: 10.1159/000078188. PMID: 15237206.

Ku J.-C., Ronceret A., Golubovskaya I., Lee D.H., Wang C., Timofejeva L., Kao Y.H., Gomez Angoa A-K., Kremling K., Williams-Carrier R., Meeley R., Barkan A., Cande W.Z., Wang C,R. 2020. Dynamic localization of SPO11-1 and conformational changes of meiotic axial elements during recombination initiation of maize meiosis. ***PLoS Genet.*** 16(4):e1007881. doi: 10.1371/journal.pgen.1007881. PMID: 32310948; PMCID: PMC7192515.

Kumar, R., Bourbon, H. M. & de Massy, B. 2010. Functional conservation of Mei4 for meiotic DNA double-strand break formation from yeasts to mice. ***Genes Dev.*** 24, 1266–1280.

Kumar R., Ghyselinck N., Ishiguro K., Watanabe Y., Kouznetsova A., Höög C., Strong E., Schimenti J., Daniel K., Toth A., de Massy B. 2015. MEI4 – a central player in the regulation of meiotic DNA double-strand break formation in the mouse. ***J Cell Sci.*** 128(9):1800-11. doi: 10.1242/jcs.165464. Epub 2015 Mar 20. PMID: 25795304; PMCID: PMC4446737.

Kumar, R., Oliver C., Brun C., Juarez-Martinez A.B., Tarabay Y., Kadlec J., de Massy B. 2018. Mouse REC114 is essential for meiotic DNA double-strand break formation and forms a complex with MEI4. ***Life Sci Alliance***. 10;1(6):e201800259. doi: 10.26508/lsa.201800259. PMID: 30569039; PMCID: PMC6288613.

Kurzbauer M-T., Janisiw M.P., Paulin L.F., Prusén Mota I., Tomanov K., Krsicka O., Haeseler A.V., Schubert V., Schlögelhofer P. 2021. ATM controls meiotic DNA double-strand break formation and recombination and affects synaptonemal complex organization in plants. ***Plant Cell.*** 33(5):1633-1656. doi: 10.1093/plcell/koab045. PMID: 33659989; PMCID: PMC8254504.

Lambing C., Osman K., Nuntasoontorn K., West A., Higgins J.D., Copenhaver G.P., Yang J., Armstrong S.J., Mechtler K., Roitinger E., Franklin FC. 2015. Arabidopsis PCH2 Mediates Meiotic Chromosome Remodeling and Maturation of Crossovers. ***PLoS Genet.*** 11(7):e1005372. doi: 10.1371/journal.pgen.1005372. PMID: 26182244; PMCID: PMC4504720.

Lambing C., Kuo P.C., Tock A.J., Topp S.D., Henderson IR. 2020. ASY1 acts as a dosage-dependent antagonist of telomere-led recombination and mediates crossover interference in *Arabidopsis*. ***Proc Natl Acad Sci U S A.*** 117(24):13647-13658. doi: 10.1073/pnas.1921055117. Epub 2020 Jun 4. PMID: 32499315; PMCID: PMC7306779.

Lambing C., Kuo P., Kim J., Osman K., Whitbread A.L., Yang J., Choi K., Franklin F.C.H, Henderson I.R. 2022. Differentiated function and localisation of SPO11-1 and PRD3 on the chromosome axis during meiotic DSB formation in *Arabidopsis thaliana*. ***PLoS Genet.*** 18(7):e1010298. doi: 10.1371/journal.pgen.1010298. PMID: 35857772; PMCID: PMC9342770.

Lange J., Pan J., Cole F., Thelen M.P., Jasin M., Keeney S. 2011. ATM controls meiotic double-strand-break formation. ***Nature.*** 479(7372):237-40. doi: 10.1038/nature10508. PMID: 22002603; PMCID: PMC3213282.

Laroussi H., Juarez-Martinez A.B., Le Roy A., Boeri Erba E., Gabel F., de Massy B., Kadlec J. 2023. Characterization of the REC114-MEI4-IHO1 complex regulating meiotic DNA double-strand break formation. ***EMBO J.*** 42(16):e113866. doi: 10.15252/embj.2023113866. Epub 2023 Jul 11. PMID: 37431931; PMCID: PMC10425845.

Lee D.H., Kao Y.H., Ku J.C., Lin C.Y., Meeley R., Jan Y.S., Wang C.J. 2015. The Axial Element Protein DESYNAPTIC2 Mediates Meiotic Double-Strand Break Formation and Synaptonemal Complex Assembly in Maize. ***Plant Cell.*** 27(9):2516-29. doi: 10.1105/tpc.15.00434. Epub 2015 Aug 21. PMID: 26296964; PMCID: PMC4815100.

Li X.C., Schimenti J.C. 2007. Mouse pachytene checkpoint 2 (trip13) is required for completing meiotic recombination but not synapsis. ***PLoS Genet.*** 3(8):e130. doi: 10.1371/journal.pgen.0030130. Epub 2007 Jun 21. Erratum in: PLoS Genet. 2007 Sep 7;3(9):e168. Li, Xin [corrected to Li, Xin Chenglin]. PMID: 17696610; PMCID: PMC1941754.

Li M., Li S., He Y., Wang Y., Zhang T., Li P., He Y. 2022. ZmSPO11-2 is critical for meiotic recombination in maize. ***Chromosome Res.*** 30(4):415-428. doi: 10.1007/s10577-022-09694-5. Epub 2022 Jun 8. PMID: 35674907.

Libby, B. J., Reinholdt, L. G., Schimenti, J. C. 2003. Positional cloning and characterization of Mei1, a vertebrate-specific gene required for normal meiotic chromosome synapsis in mice. ***Proc. Natl Acad. Sci. USA*** 100, 15706–15711.

Lin, Y., Smith, G. R. 1994. Transient, meiosis-induced expression of the rec6 and rec12 genes of *Schizosaccharomyces pombe*. ***Genetics*** 136, 769–779.

Lin, Y., Smith, G. R. 1995. An intron-containing meiosis-induced recombination gene, rec15, of *Schizosaccharomyces pombe*. ***Mol. Microbiol.*** 17, 439–448.

Liu Y., Lin Z., Yan J., Zhang X., Tong MH. 2024. A Rad50-null mutation in mouse germ cells causes reduced DSB formation, abnormal DSB end resection and complete loss of germ cells. ***Development.*** 151(8):dev202312. doi: 10.1242/dev.202312. Epub 2024 Apr 16. PMID: 38512324.

Malone R.E., Bullard S., Hermiston M., Rieger R., Cool M., Galbraith A. 1991. Isolation of mutants defective in early steps of meiotic recombination in the yeast *Saccharomyces cerevisiae*. ***Genetics.*** 128(1):79-88. doi: 10.1093/genetics/128.1.79. PMID: 2060778; PMCID: PMC1204456.

Menees, T. M., Roeder, G. S. 1989. MEI4, a yeast gene required for meiotic recombination. ***Genetics*** 123, 675–682.

Miao C., Tang D., Zhang H., Wang M., Li Y., Tang S., Yu H., Gu M., Cheng Z. 2013. Central region component1, a novel synaptonemal complex component, is essential for meiotic recombination initiation in rice. ***Plant Cell.*** 25(8):2998-3009. doi: 10.1105/tpc.113.113175. Epub 2013 Aug 13. PMID: 23943860; PMCID: PMC3784594.

Milano C.R., Ur SN., Gu Y., Zhang J., Allison R., Brown G., Neale MJ., Tromer EC., Corbett KD., Hochwagen A. 2024. Chromatin binding by HORMAD proteins regulates meiotic recombination initiation. ***EMBO J.*** 43(5):836-867. doi: 10.1038/s44318-024-00034-3. Epub 2024 Feb 8. PMID: 38332377; PMCID: PMC10907721.

Miyoshi T., Ito M., Kugou K., Yamada S., Furuichi M., Oda A., Yamada T., Hirota K., Masai H., Ohta K. 2012. A central coupler for recombination initiation linking chromosome architecture to S phase checkpoint. ***Mol Cell.*** 47(5):722-33. doi: 10.1016/j.molcel.2012.06.023. Epub 2012 Jul 26. PMID: 22841486.

Molnar M., Parisi S., Kakihara Y., Nojima H., Yamamoto A., Hiraoka Y., Bozsik A, Sipiczki M, Kohli J. 2001. Characterization of rec7, an early meiotic recombination gene in *Schizosaccharomyces pombe*. ***Genetics*** 157(2):519-32. doi: 10.1093/genetics/157.2.519. PMID: 11156975; PMCID: PMC1461520.

Murakami H., Lam I., Huang PC., Song J., van Overbeek M., Keeney S. 2020. Multilayered mechanisms ensure that short chromosomes recombine in meiosis. ***Nature.*** 582(7810):124-128. doi: 10.1038/s41586-020-2248-2. Epub 2020 May 6. PMID: 32494071; PMCID: PMC7298877.

Nonomura K., Nakano M., Fukuda T., Eiguchi M., Miyao A., Hirochika H., Kurata N. 2004. The novel gene HOMOLOGOUS PAIRING ABERRATION IN RICE MEIOSIS1 of rice encodes a putative coiled-coil protein required for homologous chromosome pairing in meiosis. ***Plant Cell.*** 16(4):1008-20. doi: 10.1105/tpc.020701. Epub 2004 Mar 18. PMID: 15031413; PMCID: PMC412873.

Nonomura K.I., Nakano M., Murata K., Miyoshi K., Eiguchi M., Miyao A., Hirochika H., Kurata N. 2004. An insertional mutation in the rice PAIR2 gene, the ortholog of Arabidopsis ASY1, results in a defect in homologous chromosome pairing during meiosis. ***Mol Genet Genomics.*** 271(2):121-9. doi: 10.1007/s00438-003-0934-z. Epub 2004 Jan 31. PMID: 14758540.

Nonomura K., Nakano M., Eiguchi M., Suzuki T., Kurata N. 2006. PAIR2 is essential for homologous chromosome synapsis in rice meiosis I. ***J Cell Sci.*** 119(Pt 2):217-25. doi: 10.1242/jcs.02736. PMID: 16410547.

Nore A., Juarez-Martinez AB., Clément J., Brun C., Diagouraga B., Laroussi H., Grey C., Bourbon HM., Kadlec J., Robert T., de Massy B. 2022. TOPOVIBL-REC114 interaction regulates meiotic DNA double-strand breaks. ***Nat Commun.*** 13(1):7048. doi: 10.1038/s41467-022-34799-0. PMID: 36396648; PMCID: PMC9671922.

Pacheco S., Maldonado-Linares A., Marcet-Ortega M., Rojas C., Martínez-Marchal A., Fuentes-Lazaro J., Lange J., Jasin M., Keeney S, Fernández-Capetillo O., Garcia-Caldés M., Roig I. 2018. ATR is required to complete meiotic recombination in mice. ***Nat Commun.*** 9(1):2622. doi: 10.1038/s41467-018-04851-z. PMID: 29977027; PMCID: PMC6033890.

Papanikos F., Clément J.A.J., Testa E., Ravindranathan R., Grey C., Dereli I., Bondarieva A., Valerio-Cabrera S., Stanzione M., Schleiffer A., Jansa P., Lustyk D., Fei J.F., Adams I.R., Forejt J., Barchi M., de Massy B., Toth A. 2019. Mouse ANKRD31 Regulates Spatiotemporal Patterning of Meiotic Recombination Initiation and Ensures Recombination between X and Y Sex Chromosomes. ***Mol Cell.*** 74(5):1069-1085.e11. doi: 10.1016/j.molcel.2019.03.022. Epub 2019 Apr 15. PMID: 31000436.

Pawlowski W.P., Golubovskaya I.N., Timofejeva L., Meeley R.B., Sheridan W.F., Cande W.Z. 2004. Coordination of meiotic recombination, pairing, and synapsis by PHS1. ***Science.*** 303(5654):89-92. doi: 10.1126/science.1091110. PMID: 14704428.

Pedroza-Garcia J.A., Eekhout T., Achon I., Nisa M.U., Coussens G., Vercauteren I., Van den Daele H., Pauwels L., Van Lijsebettens M., Raynaud C., De Veylder L. 2021. Maize ATR safeguards genome stability during kernel development to prevent early endosperm endocycle onset and cell death. ***Plant Cell.*** 33(8):2662-2684. doi: 10.1093/plcell/koab158. PMID: 34086963; PMCID: PMC8408457.

Pochon G., Henry IM., Yang C., Lory N., Fernández-Jiménez N., Böwer F., Hu B., Carstens L., Tsai HT., Pradillo M., Comai L., Schnittger A. 2022. The *Arabidopsis* Hop1 homolog ASY1 mediates cross-over assurance and interference. ***PNAS Nexus.*** 2(3):pgac302. doi: 10.1093/pnasnexus/pgac302. PMID: 36992817; PMCID: PMC10042279.

Puizina, J., Siroky, J., Mokros, P., Schweizer, D., Riha, K. 2004. Mre11 deficiency in Arabidopsis is associated with chromosomal instability in somatic cells and Spo11-dependent genome fragmentation during meiosis. ***Plant. Cell*** 16, 1968–1978.

Robert T., Nore A., Brun C., Maffre C., Crimi B., Bourbon H.M., de Massy B. 2016. The TopoVIB-Like protein family is required for meiotic DNA double-strand break formation. ***Science.*** 351(6276):943-9. doi: 10.1126/science.aad5309. Erratum in: Science. 2016 May 6;352(6286):aaf9649. doi: 10.1126/science.aaf9649. PMID: 26917764.

Rockmill, B., Roeder, G. S. 1990. Meiosis in asynaptic yeast. ***Genetics*** 126, 563–574.

Roig I., Dowdle J.A., Toth A., de Rooij D.G., Jasin M., Keeney S. 2010. Mouse TRIP13/PCH2 is required for recombination and normal higher-order chromosome structure during meiosis. ***PLoS Genet.*** 6(8):e1001062. doi: 10.1371/journal.pgen.1001062. PMID: 20711356; PMCID: PMC2920839.

Romanienko, P. J., Camerini-Otero, R. D. 2000. The mouse *Spo11* gene is required for meiotic chromosome synapsis. ***Mol. Cell*** 6, 975–987.

Ronceret, A., Doutriaux, M.-P., Golubovskaya, I. N., Pawlowski, W. P. 2009. PHS1 regulates meiotic recombination and homologous chromosome pairing by controlling the transport of RAD50 to the nucleus. ***Proc. Natl Acad. Sci. USA*** 106, 20121–20126.

San-Segundo P.A., Roeder G.S. 1999. Pch2 links chromatin silencing to meiotic checkpoint control. ***Cell.*** 97(3):313-24. doi: 10.1016/s0092-8674(00)80741-2. PMID: 10319812.

Sanchez-Moran E., Santos J.L., Jones G.H., Franklin FC. 2007. ASY1 mediates AtDMC1-dependent interhomolog recombination during meiosis in Arabidopsis. ***Genes Dev.*** 21(17):2220-33. doi: 10.1101/gad.439007. PMID: 17785529; PMCID: PMC1950860.

Schuyler S.C., Wu Y.O., Chen H.Y., Ding Y.S., Lin C.J., Chu Y.T., Chen T.C., Liao L, Tsai W.W., Huang A., Wang L.I., Liao T.W., Jhuo J.H., Cheng V. 2018. Peptide inhibitors of the anaphase promoting-complex that cause sensitivity to microtubule poison. ***PLoS One*** 13(6):e0198930. doi: 10.1371/journal.pone.0198930. PMID: 29883473; PMCID: PMC5993284.

Shi W., Ji J., Xue Z., Zhang F., Miao Y., Yang H., Tang D., Du G., Li Y., Shen Y., Cheng Z. 2021. PRD1, a homologous recombination initiation factor, is involved in spindle assembly in rice meiosis. ***New Phytol.*** 230(2):585-600. doi: 10.1111/nph.17178. Epub 2021 Feb 10. PMID: 33421144.

Shin Y.H., Choi Y., Erdin S.U., Yatsenko S.A., Kloc M., Yang F., Wang P.J., Meistrich M.L., Rajkovic A. 2010. Hormad1 mutation disrupts synaptonemal complex formation, recombination, and chromosome segregation in mammalian meiosis. ***PLoS Genet.*** 6(11):e1001190. doi: 10.1371/journal.pgen.1001190. Erratum in: PLoS Genet. 2011;7(2). doi: 10.1371/annotation/8aa656b6-55f7-4795-a441-cf243ea62175. PMID: 21079677; PMCID: PMC2973818.

Stacey N.J., Kuromori T., Azumi Y., Roberts G., Breuer C., Wada T., Maxwell A., Roberts K., Sugimoto-Shirasu K. 2006. Arabidopsis SPO11-2 functions with SPO11-1 in meiotic recombination. ***Plant J.*** 48(2):206-16. doi: 10.1111/j.1365-313X.2006.02867.x. PMID: 17018031.

Stanzione M., Baumann M., Papanikos F., Dereli I., Lange J., Ramlal A., Tränkner D., Shibuya H., de Massy B., Watanabe Y., Jasin M., Keeney S., Tóth A. 2016. Meiotic DNA break formation requires the unsynapsed chromosome axis-binding protein IHO1 (CCDC36) in mice. ***Nat Cell Biol.*** 18(11):1208-1220. doi: 10.1038/ncb3417. Epub 2016 Oct 10. PMID: 27723721; PMCID: PMC5089853.

Steiner, S., Kohli, J., Ludin, K. 2010. Functional interactions among members of the meiotic initiation complex in fission yeast. ***Curr. Genet.*** 56, 237–249.

Tang Y., Yin Z., Zeng Y., Zhang Q., Chen L., He Y., Lu P., Ye D., Zhang X. 2017. MTOPVIB interacts with AtPRD1 and plays important roles in formation of meiotic DNA double-strand breaks in Arabidopsis. ***Sci Rep.*** 7(1):10007. doi: 10.1038/s41598-017-10270-9. PMID: 28855712; PMCID: PMC5577129.

Tavassoli, M., Shayeghi, M., Nasim, A. & Watts, F. Z. 1995. Cloning and characterisation of the *Schizosaccharomyces pombe* rad32 gene: a gene required for repair of double strand breaks and recombination. ***Nucleic Acids Res.*** 23, 383–388.

Thompson, E. A. & Roeder, G. S. 1989. Expression and DNA sequence of RED1, a gene required for meiosis I chromosome segregation in yeast. ***Mol. Gen. Genet.*** 218, 293–301.

Usui T., Ogawa H., Petrini J.H. 2001. A DNA damage response pathway controlled by Tel1 and the Mre11 complex. ***Mol Cell.*** 7(6):1255-66. doi: 10.1016/s1097-2765(01)00270-2. PMID: 11430828.

Vrielynck N., Chambon A., Vezon D., Pereira L., Chelysheva L., De Muyt A., Mézard C., Mayer C., Grelon M. 2016. A DNA topoisomerase VI-like complex initiates meiotic recombination. ***Science.*** 351(6276):939-43. doi: 10.1126/science.aad5196. PMID: 26917763.

Vrielynck N., Schneider K., Rodriguez M., Sims J., Chambon A., Hurel A., De Muyt A., Ronceret A., Krsicka O, Mézard C., Schlögelhofer P., Grelon M. 2021. Conservation and divergence of meiotic DNA double strand break forming mechanisms in *Arabidopsis thaliana*. ***Nucleic Acids Res.*** 49(17):9821-9835. doi: 10.1093/nar/gkab715. PMID: 34458909; PMCID: PMC8464057.

Walker J., Gao H., Zhang J., Aldridge B., Vickers M., Higgins J.D., Feng X. 2018. Sexual-lineage-specific DNA methylation regulates meiosis in Arabidopsis. ***Nat Genet.*** 50(1):130-137. doi: 10.1038/s41588-017-0008-5. Epub 2017 Dec 18. PMID: 29255257; PMCID: PMC7611288.

Wang C., Qu S., Zhang J., Fu M., Chen X., Liang W. 2023. OsPRD2 is essential for double-strand break formation, but not spindle assembly during rice meiosis. ***Front Plant Sci.*** 13:1122202. doi: 10.3389/fpls.2022.1122202. PMID: 36714725; PMCID: PMC9880466.

Wang K., Wang M., Tang D., Shen Y., Qin B., Li M., Cheng Z. 2011. PAIR3, an axis-associated protein, is essential for the recruitment of recombination elements onto meiotic chromosomes in rice. ***Mol Biol Cell.*** 22(1):12-9. doi: 10.1091/mbc.E10-08-0667. Epub 2010 Nov 30. PMID: 21119003; PMCID: PMC3016970.

Wang Y., Wang Y., Zang J., Chen H., He Y. 2022. ZmPRD1 is essential for double-strand break formation, but is not required for bipolar spindle assembly during maize meiosis. ***J Exp Bot.*** 73(11):3386-3400. doi: 10.1093/jxb/erac075. PMID: 35201286.

Wang Y., Li S.Y., Wang Y.Z., He Y. 2023. ZmASY1 interacts with ZmPRD3 and is crucial for meiotic double-strand break formation in maize. ***New Phytol.*** 237(2):454-470. doi: 10.1111/nph.18528. Epub 2022 Nov 30. PMID: 36221195.

Waterworth W.M., Altun C., Armstrong S.J., Roberts N., Dean P.J., Young K., Weil C.F., Bray C.M., West C.E. 2007. NBS1 is involved in DNA repair and plays a synergistic role with ATM in mediating meiotic homologous recombination in plants. ***Plant J.*** 52(1):41-52. doi: 10.1111/j.1365-313X.2007.03220.x. Epub 2007 Aug 2. PMID: 17672843.

Widger A., Mahadevaiah S.K., Lange J., ElInati E., Zohren J., Hirota T., Pacheco S., Maldonado-Linares A., Stanzione M., Ojarikre O., Maciulyte V., de Rooij D.G., Tóth A., Roig I., Keeney S., Turner JMA. 2018. ATR is a multifunctional regulator of male mouse meiosis. ***Nat Commun.*** 9(1):2621. doi: 10.1038/s41467-018-04850-0. PMID: 29976923; PMCID: PMC6033951.

Wojtasz L., Daniel K., Roig I., Bolcun-Filas E., Xu H., Boonsanay V., Eckmann C.R., Cooke H.J., Jasin M., Keeney S., McKay M.J., Toth A. 2009. Mouse HORMAD1 and HORMAD2, two conserved meiotic chromosomal proteins, are depleted from synapsed chromosome axes with the help of TRIP13 AAA-ATPase. ***PLoS Genet.*** 5(10):e1000702. doi: 10.1371/journal.pgen.1000702. Epub 2009 Oct 23. PMID: 19851446; PMCID: PMC2758600.

Xu Y., Ashley T., Brainerd E.E., Bronson R.T., Meyn M.S., Baltimore D. 1996. Targeted disruption of ATM leads to growth retardation, chromosomal fragmentation during meiosis, immune defects, and thymic lymphoma. ***Genes Dev.*** 10(19):2411-22. doi: 10.1101/gad.10.19.2411. PMID: 8843194.

Xu J., Li T., Kim S., Boekhout M., Keeney S. 2023. Essential roles of the ANKRD31-REC114 interaction in meiotic recombination and mouse spermatogenesis. ***Proc Natl Acad Sci U S A.*** 120(47):e2310951120. doi: 10.1073/pnas.2310951120. Epub 2023 Nov 17. PMID: 37976262; PMCID: PMC10666023.

Xue Z., Li Y., Zhang L., Shi W., Zhang C., Feng M., Zhang F., Tang D., Yu H., Gu M., Cheng Z. 2016. OsMTOPVIB Promotes Meiotic DNA Double-Strand Break Formation in Rice. ***Mol Plant.*** 9(11):1535-1538. doi: 10.1016/j.molp.2016.07.005. Epub 2016 Jul 22. PMID: 27456761.

Xue Z., Liu C., Shi W., Miao Y., Shen Y., Tang D., Li Y., You A., Xu Y., Chong K., Cheng Z. 2019. OsMTOPVIB is required for meiotic bipolar spindle assembly. ***Proc Natl Acad Sci U S A.*** 116(32):15967-15972. doi: 10.1073/pnas.1821315116. Epub 2019 Jul 24. PMID: 31341087; PMCID: PMC6689953.

Yang C., Hu B., Portheine S.M., Chuenban P., Schnittger A. 2020. State changes of the HORMA protein ASY1 are mediated by an interplay between its closure motif and PCH2. ***Nucleic Acids Res.*** 48(20):11521-11535. doi: 10.1093/nar/gkaa527. PMID: 32558910; PMCID: PMC7672429.

Yin Y., Cheong H., Friedrichsen D., Zhao Y., Hu J., Mora-Garcia S., Chory J. 2002. A crucial role for the putative Arabidopsis topoisomerase VI in plant growth and development. ***Proc Natl Acad Sci U S A.*** 99(15):10191-6. doi: 10.1073/pnas.152337599. Epub 2002 Jul 15. PMID: 12119417; PMCID: PMC126646.

Young, J. A., Hyppa, R. W. & Smith, G. R. 2004. Conserved and nonconserved proteins for meiotic DNA breakage and repair in yeasts. ***Genetics*** 167, 593–605.

Yu H., Wang M., Tang D., Wang K., Chen F., Gong Z., Gu M., Cheng Z. 2010. OsSPO11-1 is essential for both homologous chromosome pairing and crossover formation in rice. ***Chromosoma.*** 119(6):625-36. doi: 10.1007/s00412-010-0284-7. Epub 2010 Jul 13. PMID: 20625906.

Yu H., Zhang L., He X., Zhang T., Wang C., Lu J., He X., Chen K., Gu W., Cheng S., Hu Y., Yao B., Jian A., Yu X., Zheng H., You S., Wang Q., Lei D., Jiang L., Zhao Z., Wan J. 2022. OsPHS1 is required for both male and female gamete development in rice. ***Plant Sci.*** 325:111480. doi: 10.1016/j.plantsci.2022.111480. Epub 2022 Sep 29. PMID: 36183810.

Yuan W., Li X., Chang Y., Wen R., Chen G., Zhang Q., Wu C. 2009. Mutation of the rice gene PAIR3 results in lack of bivalent formation in meiosis. ***Plant J.*** 59(2):303-15. doi: 10.1111/j.1365-313X.2009.03870.x. Epub 2009 Apr 14. PMID: 19392701.

Zhang, B., Tang, Z., Li, L. & Lu, L. Y. 2020. NBS1 is required for SPO11-linked DNA double-strand break repair in male meiosis. ***Cell Death Differ.*** 27, 2176–2190.

Zhang C., Song Y., Cheng Z.H., Wang Y.X., Zhu J., Ma H., Xu L., Yang Z.N. 2012. The *Arabidopsis thaliana* DSB formation (AtDFO) gene is required for meiotic double-strand break formation. ***Plant J.*** 72(2):271-81. doi: 10.1111/j.1365-313X.2012.05075.x. Epub 2012 Jul 26. PMID: 22694475.

Zhang C., Zhang F., Cheng X., Liu K., Tang J., Li Y., Tang D., Cheng Z., Yu H. 2020. OsATM Safeguards Accurate Repair of Meiotic Double-Strand Breaks in Rice. ***Plant Physiol.*** 183(3):1047-1057. doi: 10.1104/pp.20.00053. Epub 2020 May 13. PMID: 32404412; PMCID: PMC7333689.

Zhang, L., Kim, K.P., Kleckner, N.E., Storlazzi, A. 2011. Meiotic double-strand breaks occur once per pair of (sister) chromatids and, via Mec1/ATR and Tel1/ATM, once per quartet of chromatids. ***Proc Natl Acad Sci U S A.*** 108(50):20036-41. doi: 10.1073/pnas.1117937108. Epub 2011 Nov 28. Erratum in: Proc Natl Acad Sci U S A. 2012 Jan 24;109(4):1353. PMID: 22123968; PMCID: PMC3250133.

Zhao J., Gui X., Ren Z., Fu H., Yang C., Wang W., Liu Q., Zhang M., Wang C., Schnittger A., Liu B. 2023. ATM-mediated double-strand break repair is required for meiotic genome stability at high temperature. ***Plant J.*** 114(2):403-423. doi: 10.1111/tpj.16145. Epub 2023 Mar 3. PMID: 36786716.

Zhou L., Han J., Chen Y., Wang Y., Liu YG. 2017. Bivalent Formation 1, a plant-conserved gene, encodes an OmpH/coiled-coil motif-containing protein required for meiotic recombination in rice. ***J Exp Bot.*** 68(9):2163-2174. doi: 10.1093/jxb/erx077. PMID: 28369589; PMCID: PMC5447885.
